# Lactate-Induced Mitochondrial Calcium Uptake 3 Aggravates Myocardial Ischemia–Reperfusion Injury by Promoting Neutrophil Extracellular Trap Formation

**DOI:** 10.34133/research.0705

**Published:** 2025-05-30

**Authors:** Hongru Zhang, Lei Liu, Chuchu Shen, Xinxue Jiang, Jing Liu, Jing Chen, Senlei Xu, Yanfei Mo

**Affiliations:** ^1^ School of Acupuncture and Tuina, School of Regimen and Rehabilitation, Nanjing University of Chinese Medicine, Nanjing, China.; ^2^ Dan’an College, Nanjing University of Chinese Medicine, Nanjing, China.; ^3^ Department of Ultrasound Medicine, Affiliated Drum Tower Hospital of Nanjing University Medical School, Nanjing, China.; ^4^ Department of Cardiology, Pukou Hospital of Chinese Medicine Affiliated to China Pharmaceutical University, Nanjing, China.; ^5^ School of Medicine, Nanjing University of Chinese Medicine, Nanjing, China.

## Abstract

**Background:** Ischemic heart disease is a leading cause of mortality and disability worldwide among cardiovascular conditions. Myocardial ischemia–reperfusion injury (MIRI) occurs following percutaneous coronary intervention, during which neutrophils generate neutrophil extracellular traps (NETs) in response to injury. This study aims to elucidate the mechanisms underlying NET activation and its impact on MIRI. **Methods:** Sham and MIRI rat models were established. Various techniques, including enzyme-linked immunosorbent assay, hematoxylin and eosin staining, Masson staining, and transmission electron microscopy, were used to assess endothelial cell injury and myocardial tissue inflammation. Immunofluorescence was employed to evaluate NET activation in tissues, peripheral blood neutrophils, and protein colocalization. MitoTracker and ER-Tracker staining were conducted to assess the formation of mitochondria-associated membranes (MAMs). Extracted NETs were applied to conduct microvascular endothelial cell tube formation assay and flow cytometry. RNA-sequencing and immunoprecipitation–mass spectrometry were applied to determine the key regulators. Flow cytometry and Western blot were used to assess Ca^2+^ and mitophagy levels in neutrophils. Deoxyribonuclease I, NET inhibitor, was injected into MIRI rats to evaluate the in vivo effects of NET modulation on MIRI severity. **Results:** MIRI was often accompanied by cardiac microvascular endothelial cell (CMEC) injury and inflammation. Lactate mediated H3K18 lactylation at the MICU3 promoter in neutrophils, enhancing its transcription and leading to elevated MICU3 levels. Besides, lactate also promoted the interaction between MICU3 and AASR1, stabilizing MICU3 through lactylation. Up-regulated MICU3 interacted with VDAC1, facilitating MAM formation, excessive Ca^2+^ uptake, mitochondrial dysfunction, mitophagy activation, and NET activation. Elevated NET level exacerbated CMEC dysfunction, further aggravating MIRI. **Conclusion:** Lactate-driven MICU3 transcriptional activation and stabilization facilitates NET formation, contributing to MIRI development.

## Introduction

Ischemic heart disease (IHD) remains one of the leading causes of mortality and disability worldwide [1]. As a common cardiovascular disease, IHD primarily manifests as angina pectoris and myocardial ischemia. While acute and chronic coronary syndromes are often considered primary etiological factors, the pathophysiology of IHD is complex, involving multiple processes such as inflammation, coronary microvascular dysfunction, endothelial dysfunction, thrombosis, and angiogenesis [2,3]. Additionally, the increasing prevalence of IHD is driven by well-recognized risk factors, including hypertension, smoking, obesity, and diabetes [4].

IHD is commonly managed through 3 main treatment strategies: medication therapy (MT), percutaneous coronary intervention (PCI), and coronary artery bypass grafting (CABG) [5]. However, PCI, as a form of reperfusion therapy, can be considered a double-edged sword. While PCI and other reperfusion therapies restore myocardial blood supply, they may also induce coronary microvascular dysfunction of the coronary microvasculature and trigger a series of complex pathophysiological responses, including oxidative stress, inflammatory responses, calcium overload, and mitochondrial dysfunction. These reactions can lead to further damage, collectively referred to myocardial ischemia–reperfusion injury (MIRI) [6].

Mitochondria-associated membranes (MAMs) are specialized subcellular structures formed by the close contact between the endoplasmic reticulum (ER) and mitochondria. These dynamic interfaces play a critical role in cellular homeostasis by regulating calcium ion (Ca^2+^) transfer, lipid metabolism, and mitochondrial function [7]. Dysregulation of MAMs has been implicated in various cardiovascular diseases, including MIRI. During ischemia–reperfusion, excessive Ca^2+^ transfer through MAMs can lead to mitochondrial calcium overload, triggering mitochondrial dysfunction, oxidative stress, and cell death [8]. Emerging evidence suggests that MAMs are not only structural platforms for Ca^2+^ exchange but also hubs for signaling pathways that mediate inflammation and cell survival [9]. However, the precise role of MAMs in neutrophil activation and their contribution to MIRI remain poorly understood.

Neutrophils, as an important subset of leukocytes, possess antimicrobial functions such as phagocytosis, degranulation, and the formation of neutrophil extracellular traps (NETs). In response to infection and tissue damage, neutrophils migrate from the bloodstream to inflamed tissues [10,11]. They are also critically involved in the pathology of the initiation and progression of MIRI. An existing study has shown that neutrophils infiltrate the damaged area in large numbers during reperfusion, and their excessive activation and infiltration can exacerbate MIRI [12]. Furthermore, while NETs contribute to the inflammatory response and bacterial clearance by capturing pathogens and releasing inflammatory mediators, excessive activation of NETs can promote tissue damage and organ dysfunction [13]. However, research on the activation of NETs and their specific mechanisms of action in MIRI remains currently limited and requires further exploration.

This study aims to elucidate the mechanisms underlying NET activation and its contributions to MIRI. We found that neutrophils uptake lactate, which activates the expression of the MICU3 and enhances its protein stability. MICU3 interacts with VDAC1, promoting the formation of MAMs, resulting in excessive Ca^2+^ uptake and mitochondrial dysfunction. This mitochondria dysfunction activates mitophagy, further promoting the activation of NETs, ultimately resulting in damage to microvascular endothelial cells and exacerbating MIRI. Our findings provide a deeper understanding of the specific mechanisms through which neutrophils mediate MIRI and offer new perspectives and potential therapeutic approaches for interventions in MIRI.

## Results

### MIRI process is accompanied by myocardial microvascular endothelial cell injury and inflammatory infiltration

**Fig. 1.**
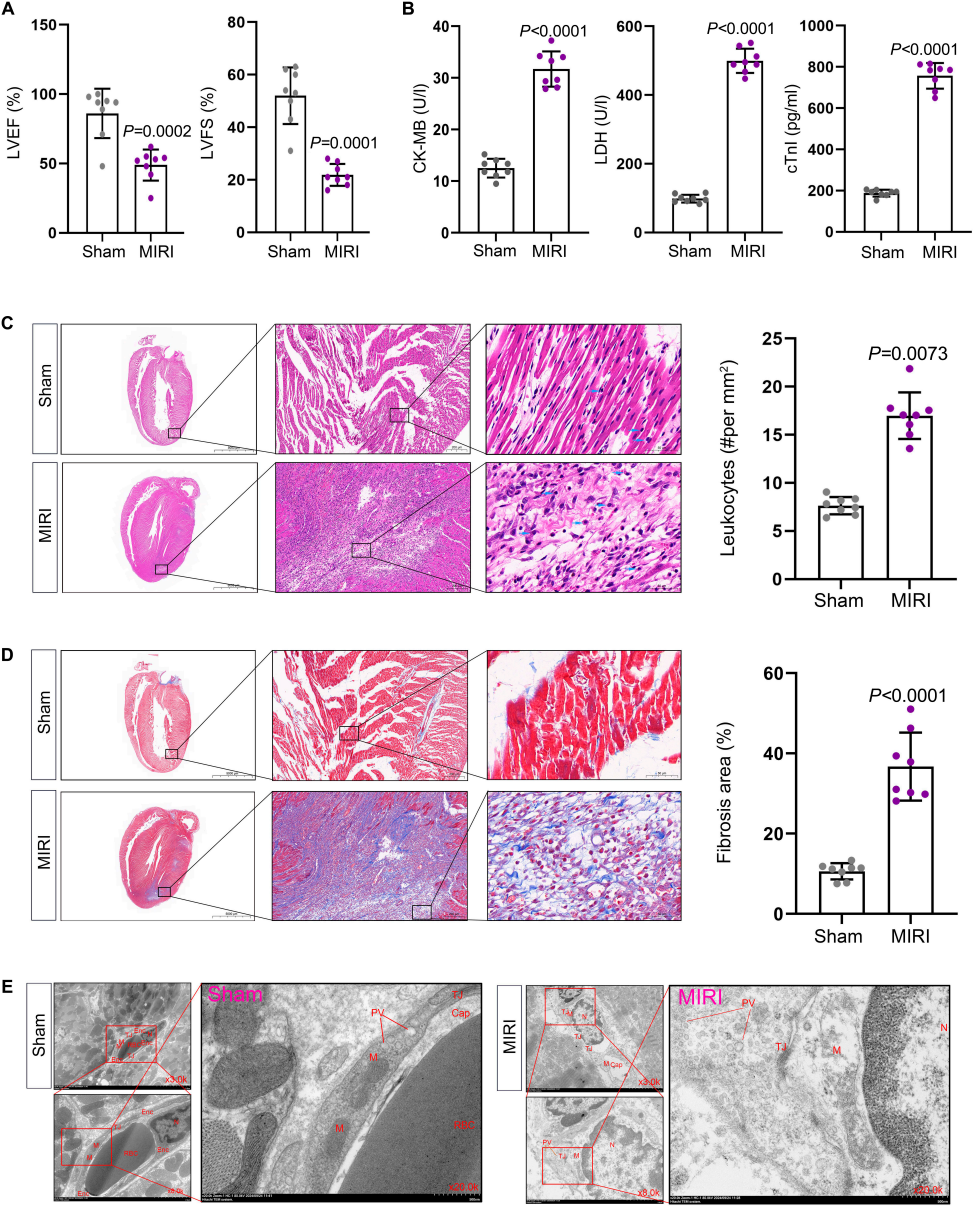
MIRI process is accompanied by CMEC injury and inflammatory infiltration. (A) The changes in cardiac function in different models (Sham and MIRI) were assessed using echocardiography. LVEF: Sham: 86.13 ± 17.84, MIRI: 48.88 ± 11.22; LVFS: Sham: 52.29 ± 10.80, MIRI: 22.00 ± 4.20 (*n* = 8 per group, mean ± SD). (B) The levels of CK-MB, LDH, and cTnI biomarkers in the serum from different groups were detected using ELISA. CK-MB: Sham: 12.47 ± 1.85 U/l, MIRI: 31.80 ± 3.44 U/l; LDH: Sham: 499.4 ± 35.30 U/l, MIRI: 31.80 ± 3.44 U/l; cTnI: Sham: 187.94 ± 16.96 pg/ml, MIRI: 756.84 ± 62.11 pg/ml (*n* = 8 per group, mean ± SD). (C) The extent of inflammatory infiltration was evaluated using H&E staining. Sham: 7.63% ± 0.89, MIRI: 16.99% ± 2.42 (*n* = 8 per group, mean ± SD). (D) Fibrosis in myocardial tissue was detected using Masson staining. Sham: 10.59% ± 2.02, MIRI: 36.71% ± 8.47 (*n* = 8 per group, mean ± SD). (E) The microvascular structure of myocardial tissue was observed using TEM (*n* = 3). Cap, vascular lumen; Enc, endothelial cells; N, nucleus; M, mitochondria; PV, pinocytotic vesicles; TJ, tight junctions.

We established a MIRI model in Sprague–Dawley rats and assessed changes in cardiac function using echocardiography, confirming the successful implementation of the model (Fig. 1A). Serum levels of myocardial enzymes, including creatine kinase-myocardial band (CK-MB), lactate dehydrogenase (LDH), and cardiac troponin I (cTnI), were quantified with enzyme-linked immunosorbent assay (ELISA), showing significantly increased levels in the MIRI group (Fig. 1B). Additionally, we performed pathological examinations on myocardial tissue samples from both the control and MIRI groups. Hematoxylin and eosin (H&E) staining revealed infiltration of inflammatory cells in the MIRI group’s myocardial tissues (Fig. 1C). Furthermore, Masson staining illustrated notable fibrosis in the MIRI group (Fig. 1D). Cardiac microvascular endothelial cells (CMECs) are vital for myocardial vascular regeneration, which are crucial for cardiac function recovery. Transmission electron microscopy (TEM) revealed that compared to the Sham group, endothelial cells in the MIRI group showed swelling, blurred cell membrane boundaries, varying sizes of vacuoles, mitochondrial swelling, multiple mitochondrial cristae fractures, and widened intercellular gaps (Fig. 1E). These findings indicate that the MIRI process is associated with damage to the cardiac microvasculature, leading to impaired barrier function of the CMECs.

### Neutrophil-derived NETs exacerbate endothelial cell injury

Neutrophils, which are abundant leukocytes in the immune system, are among the first cells to be activated following myocardial ischemia. They can migrate along the vascular endothelium in response to chemokines released from inflamed tissues and are recruited to the sites of inflammation within the myocardial tissue [14]. This process may exacerbate the inflammatory response and mediate damage to the cardiac microvasculature [15].

**Fig. 2.**
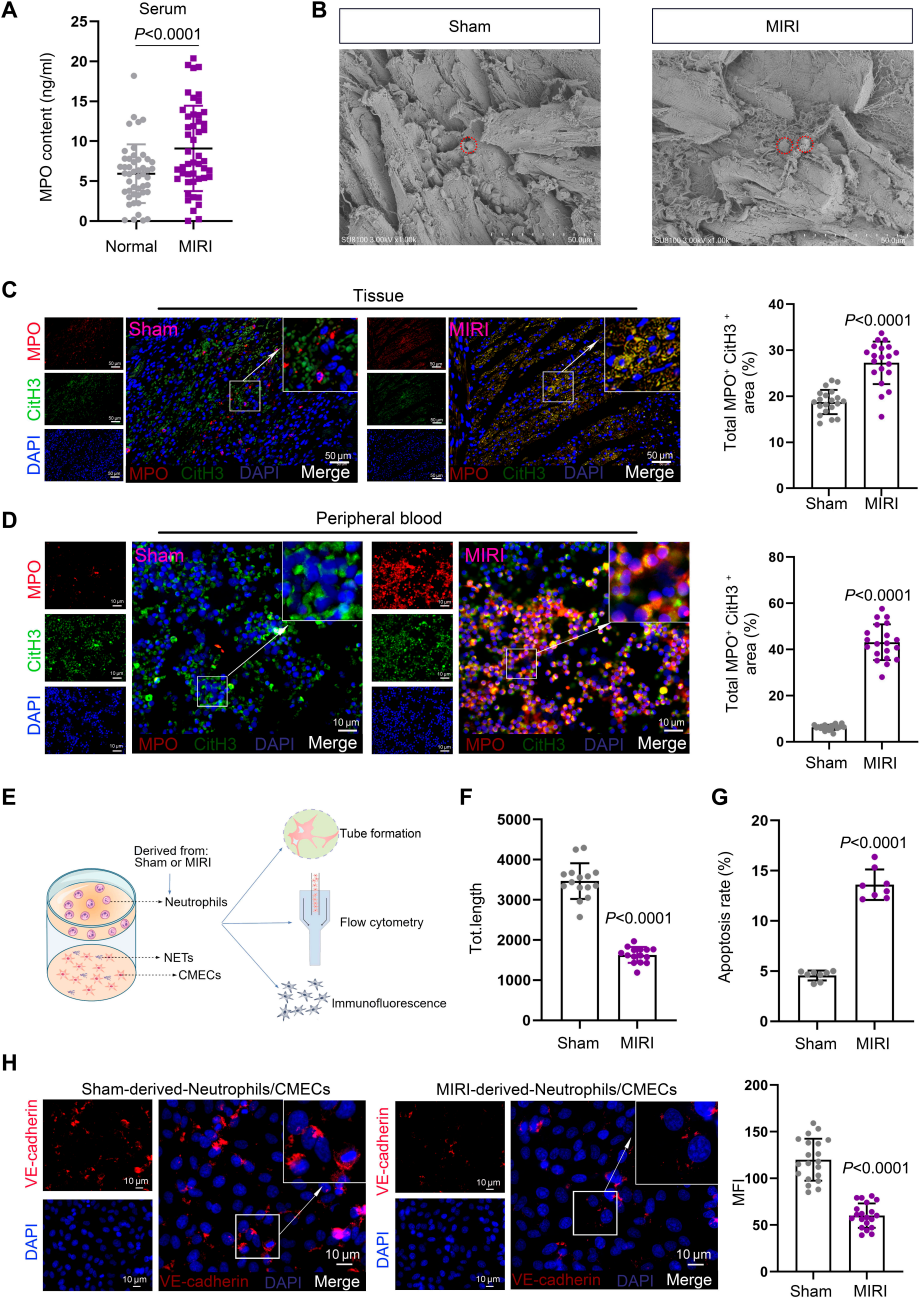
Neutrophil-derived NETs of MIRI exacerbate endothelial cell injury. (A) The MPO concentration in the serum of MIRI patients and healthy individuals was measured using ELISA (*n* = 48). (B and C) SEM and IF were performed to analyze the levels of NETs in myocardial tissues from the Sham and MIRI groups. For SEM, *n* = 3 per group; for IF (*n* = 20 filed per group): Sham: 4.33% ± 0.74%, MIRI: 18.33% ± 3.75% (mean ± SD). (D) The levels of NETs from different groups were analyzed using IF. Sham: 21.22% ± 3.57%, MIRI: 74.94% ± 8.21% (*n* = 20 filed per group, mean ± SD). (E) CMECs were cocultured with neutrophil derived from either the Sham or MIRI groups. (F) Tube formation assay was conducted to analyze the angiogenic potential of CMECs under different treatment conditions. Sham: 3,467.40 ± 442.65, MIRI: 1,627.84 ± 197.24 (*n* = 15 per group, mean ± SD). (G) Apoptosis levels in CMECs from different treatment groups were detected using flow cytometry. Sham: 4.56% ± 0.49%, MIRI: 13.61% ± 1.51% (*n* = 8 per group, mean ± SD). (H) The expression of VE-cadherin of CMECs from different treatment groups was assessed using IF. Sham: 119.95 ± 22.50, MIRI: 60.00 ± 13.10 (*n* = 20 filed per group, mean ± SD).

Based on clinical data, we observed that serum myeloperoxidase (MPO) concentration in MIRI patients was significantly higher than those in healthy individuals, indicating substantial NET formation of MIRI patients (Fig. 2A). Subsequently, we investigated the generation of NETs in the myocardial tissues of rats using scanning electron microscopy (SEM) and immunofluorescence (IF) assay with specific antibodies against MPO and citrullinated histone H3 (CiH3). The results indicated that a substantial increase of NETs was generated in the myocardial tissues of the MIRI group (Fig. 2B and C). Additionally, IF assay assessing the quantity of NETs in the peripheral blood neutrophils of different groups revealed a substantial increase of NETs in the MIRI group rats (Fig. 2D). After treating CMECs with NETs derived from the Sham and MIRI groups, we evaluated the tube formation and apoptosis levels of CMECs using tube formation assay and flow cytometry (Fig. 2E). The results demonstrated that NETs from MIRI rats inhibited tube formation ability of CMECs while promoting their apoptosis (Fig. 2F and G), indicating that the extensive activation of NETs contributes to the functional impairment of CMECs. Furthermore, IF results also showed that CMEC function was compromised (Fig. 2H).

Additionally, we further assessed the impact of NETs derived from different model rats on the tube formation capacity, apoptotic capability, and VE-cadherin level in human umbilical vein endothelial cells (HUVECs) using tube formation assay, flow cytometry for apoptosis analysis, and IF assay. Similarly, the results indicated that NETs from MIRI rats inhibited the angiogenic ability and VE-cadherin expression of HUVECs while enhancing their apoptotic capacity (Fig. S1A to C). In summary, the extensive generation of NETs exacerbates endothelial cell injury.

### MICU3-mediated mitochondrial homeostasis imbalance induces NET formation

**Fig. 3.**
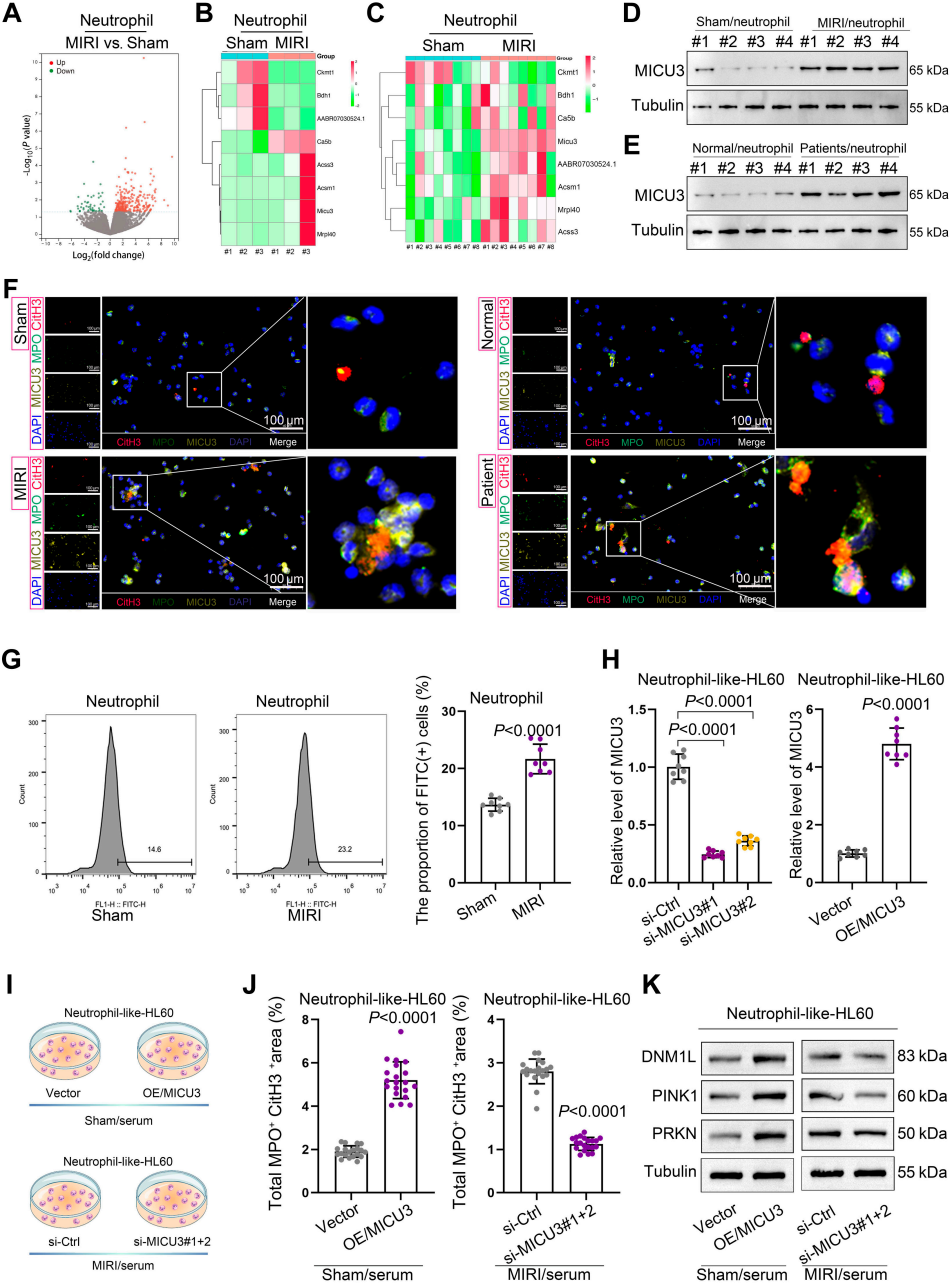
MICU3-mediated mitochondrial homeostasis imbalance induces NET formation. (A) RNA-sequencing was employed to analyze differentially expressed genes in peripheral blood neutrophils derived from the Sham and MIRI groups (*n* = 3 per group). (B) A heatmap was generated to display the expression differences of mitochondrial-related genes (Ckmt1, Bdh1, AABR07030524.1, Ca5b, Acss3, Ascm1, Micu3, and Mrpl40) in the sequencing data (*n* = 3 per group). (C) A heatmap was applied to illustrate the RT-qPCR results of mitochondrial-related gene expression in neutrophils from the Sham and MIRI groups (*n* = 8 per group). (D and E) The expression levels of MICU3 protein in neutrophils collected from different rat models and clinical patients were detected using WB (*n* = 4 per group). (F) The level of MICU3 and NETs in neutrophils collected from different rat models and clinical patients was determined by IF (*n* = 3 per group). (G) The Ca^2+^ content in neutrophils from different groups was analyzed using flow cytometry. Sham: 13.67 ± 1.12, MIRI: 21.67 ± 2.59 (*n* = 8 per group, mean ± SD). (H) MICU3 was either knocked down or overexpressed in HL60 cells, with validation performed using RT-qPCR. Knockdown: si-Ctrl: 1.01 ± 0.11, si-MICU3#1: 0.25 ± 0.03, si-MICU3#2: 0.36 ± 0.04; overexpression: Vector: 1.01 ± 0.12, OE/MICU3: 4.80 ± 0.55 (*n* = 8 per group, mean ± SD). (I) Diagrams were created to illustrate the treatment of HL60 cells (with MICU3 knockdown or overexpression) using serum from Sham or MIRI rats. (J) Quantitative analysis of IF conducted to detect the level of NET activation in HL60 cells with different treatment. Sham/serum: Vector: 1.9 ± 0.00, OE/MICU3: 50.03 ± 6.74; MIRI/serum: si-Ctrl: 26.99 ± 3.61, si-MICU3#1+2: 11.99 ± 1.49 (*n* = 20 filed per group, mean ± SD). (K) The expression differences of mitophagy proteins DNM1L, PINK1, and PRKN in HL60 cells from different treatment groups were detected using WB (*n* = 3 per group).

To investigate the reasons for the extensive activation of NETs in MIRI, we performed RNA-sequencing to analyze gene expression in peripheral blood neutrophils from the MIRI and Sham groups. This analysis identified a set of significantly dysregulated genes in MIRI-derived peripheral blood neutrophils (Fig. 3A). Previous research has demonstrated that reactive oxygen species (ROS) generated from mitochondrial dysfunction enhance mitophagy and promote NET formation [16]. Based on this clue, we hypothesized that mitochondrial dysfunction might contribute to NET formation and presented the analysis results of mitochondrial-related gene expression from the sequencing data (Fig. 3B). To identify specific genes, we conducted reverse transcription quantitative polymerase chain reaction (RT-qPCR) to measure the mitochondrial-related genes in neutrophils from different groups of rats. The results, visualized as a heatmap, revealed a significant up-regulation of MICU3 (Fig. 3C). MICU3 is an essential component of the mitochondrial calcium uniporter (MCU) complex [17]. Western blot (WB) analysis further confirmed increased MICU3 protein levels in neutrophils derived from the MIRI models and MIRI patients (Fig. 3D and E).

For further confirmation, we applied the IF to elucidate the level of MICU3 and NETs in neutrophils sourced from Sham and MIRI models, MIRI patients, and healthy individuals, and the results revealed a significant up-regulation of MICU3 and NETs (Fig. 3F). Given that MICU3 facilitates mitochondrial Ca^2+^ uptake and promotes mitochondrial ROS production [18], we assessed mitochondrial Ca^2+^ levels using flow cytometry, which revealed elevated Ca^2+^ content in neutrophils from the MIRI group (Fig. 3G). These findings suggest that the up-regulation of MICU3 promotes excessive mitochondrial Ca^2+^ uptake, leading to mitochondrial homeostasis imbalance, which is a potential cause of NET activation.

Subsequently, to validate the role of MICU3, we overexpressed or interfered with MICU3 expression in HL60 cells, a model for neutrophil-like cells (Fig. 3H). The cells were treated with serum from either Sham or MIRI groups (Fig. 3I). IF assay results indicated that overexpression of MICU3 significantly increased NET levels, while interference with MICU3 significantly reduced NET levels (Fig. 3J). Additionally, we observed that mitophagy-related proteins (DNM1L, PINK1, and PRKN) also increased with elevated MICU3 expression and decreased with reduced MICU3 expression (Fig. 3K), and the Ca^2+^ content in neutrophils followed the same trend (Fig. S1D). Furthermore, to further investigate the link between mitophagy and NET activation, we treated neutrophils from MIRI rats and MICU3-overexpressing HL60 cells with a mitophagy inhibitor [chloroquine (CQ)] while using serum (Fig. S1E). WB and IF analyses revealed that mitophagy inhibitor reduced both mitophagy and NET activation (Fig. S1F and G). These findings confirm that MICU3 up-regulation promotes NET activation through excessive mitochondrial Ca^2+^ uptake and mitophagy, establishing MICU3-mediated mitochondrial dysfunction as an important mechanism in NET activation during MIRI.

### MICU3 interacts with VDAC1, promoting the formation of MAMs to enhance mitophagy and NET activation

**Fig. 4.**
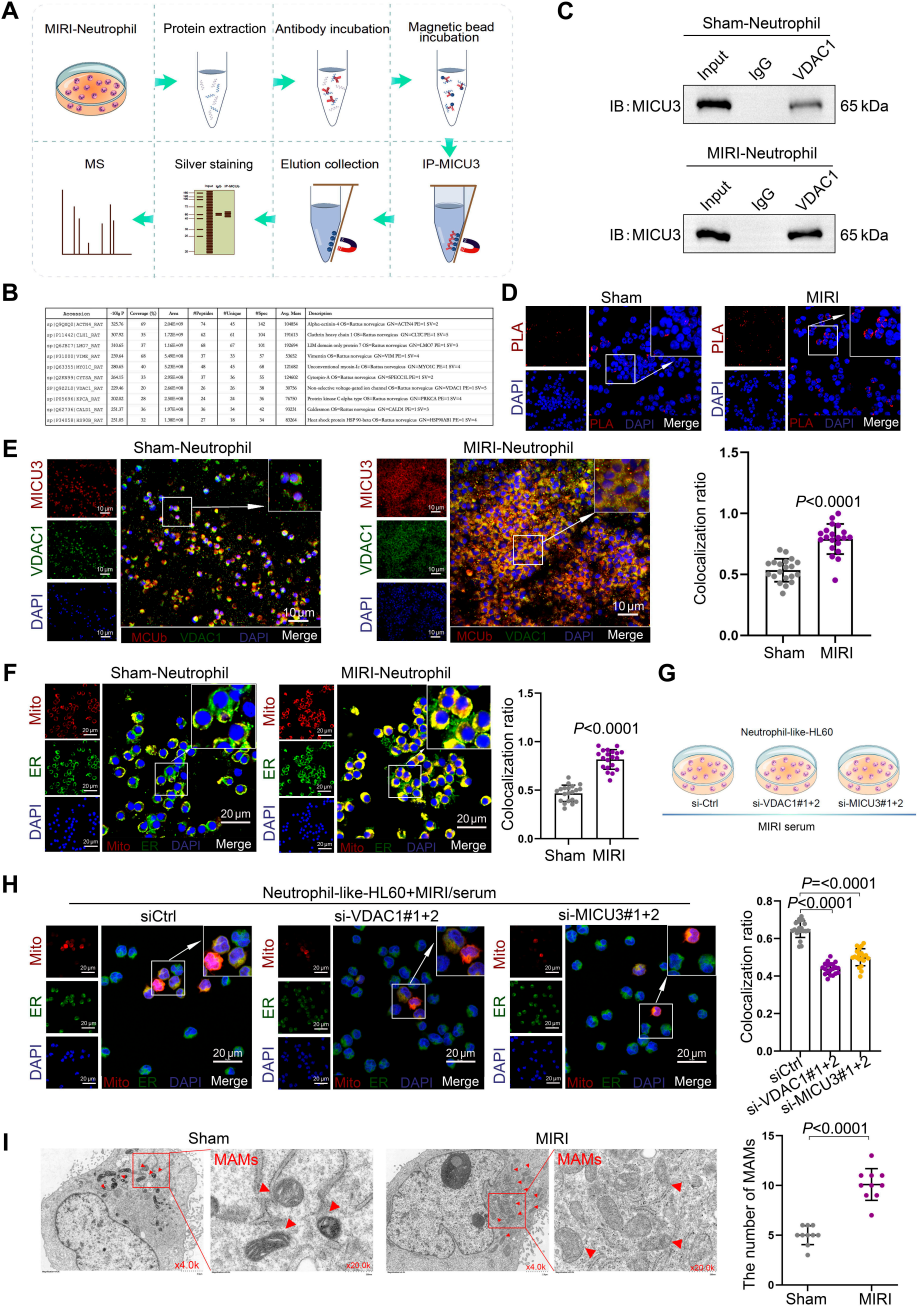
MICU3 interacts with VDAC1 promoting the formation of MAMs to enhance mitophagy and NET activation. (A) A schematic diagram was created to illustrate the binding product process obtained from the IP-MS experiment. (B) MS analysis revealed the presence of VDAC1. (C) Co-IP was performed to confirm the binding of VDAC1 to MICU3 and to assess the different binding levels in neutrophils from various groups (*n* = 3 per group). (D) PLA assay was applied to determine the combination of VDAC1 and MICU3 (*n* = 3 per group). (E) IF was conducted to detect the colocalization ratio of MICU3 and VDAC1 between the Sham and MIRI groups. Sham: 0.53 ± 0.09, MIRI: 0.79 ± 0.12 (*n* = 20 filed per group, mean ± SD). (F) MitoTracker and ER-Tracker staining was performed to analyze the differences in the number of MAMs between the Sham and MIRI groups (*n* = 20 filed per group). (G) Schematic diagram depicting HL60 cells with knockdown of VDAC1 or MICU3 treated with serum of MIRI group. (H) MitoTracker and ER-Tracker staining was conducted to assess the level of MAMs in HL60 cells that were knocked down for MICU3/VDAC1 and treated with MIRI serum. MICU3 knockdown: 0.65 ± 0.045, VDAC1 knockdown: 0.44 ± 0.03, MIRI serum: 0.50 ± 0.05 (*n* = 20 filed per group, mean ± SD). (I) SEM was applied to observe the MAMs of Sham and MIRI (*n* = 10 filed per group).

To investigate the specific mechanism by which MICU3 promotes NET activation, we designed and conducted the following experiments. Initially, we performed the immunoprecipitation (IP) experiment using MICU3 antibody in neutrophils isolated from MIRI rats to obtain its interacting proteins (Fig. 4A). Mass spectrometry (MS) analysis revealed that VDAC1 was among the interacting proteins (Fig. 4B). VDAC1 locates on the mitochondrial membrane that forms MAMs by interacting with the MCU complex [19]. This interaction mediates communication between the 2 organelles and regulates mitochondrial homeostasis [9]. We hypothesized that the interaction between MICU3 and VDAC1 promotes excessive formation of MAMs, leading to an imbalance in mitochondrial homeostasis, which subsequently triggers mitophagy and NET activation. Co-IP results further validated the binding of VDAC1 to MICU3, and observed an increased association of VDAC1 with MICU3 in neutrophils derived from MIRI, where NETs were activated (Fig. 4C). The proximity ligation assay (PLA) experiment further confirmed the interaction between VDAC1 and MICU3 (Fig. 4D). Subsequently, we analyzed the subcellular localization of MICU3 and VDAC1 in neutrophils from the Sham and MIRI groups using IF. The results confirmed that the colocalization level of MICU3 and VDAC1 was elevated in the MIRI group (Fig. 4E).

To explore the relationship between MAM and NET activation, we used MitoTracker and ER-Tracker dyes to label mitochondria and the ER, respectively. The results showed that the level of MAMs in neutrophils from MIRI rats with extensive NET activation increased significantly (Fig. 4F). We treated neutrophils extracted from peripheral blood with serum from either the Sham or MIRI group. Dual staining with MitoTracker and ER-Tracker revealed that treatment with serum from the MIRI group promoted MAM formation (Fig. S2A). Additionally, we knocked down VDAC1 (Fig. S2B) or MICU3 in HL60 cells and treated them with serum from MIRI. According to the mitochondrial and ER staining results, knockdown of MICU3 or VDAC1 inhibited MAM formation (Fig. 4G and H). Observation under a transmission electron microscope revealed that the MIRI group exhibited more MAMs (Fig. 4I). Furthermore, knockdown of VDAC1 or MICU3 also resulted in reduced Ca^2+^ content in neutrophils (Fig. S2C), as well as decreased levels of mitophagy (Fig. S2D) and NET activation (Fig. S2E).

### Circulating lactate during MIRI promotes the up-regulation of MICU3 expression

**Fig. 5.**
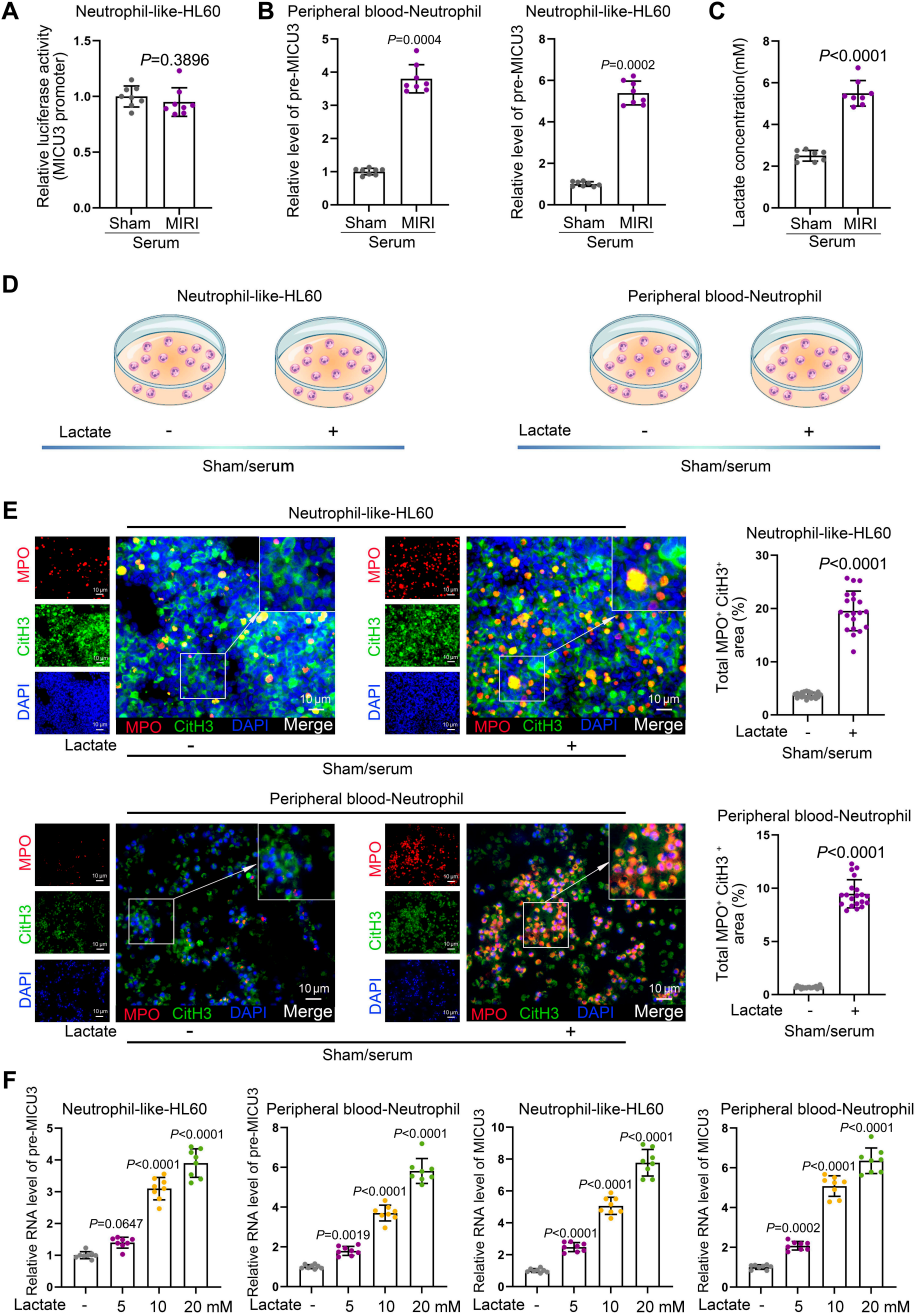
Circulating lactate during MIRI promotes the up-regulation of MICU3 expression. (A) Luciferase reporter gene assay was conducted to investigate the effects of serum from different groups on the activity of the MICU3 promoter. Sham: 1.00 ± 0.09, MIRI: 0.88 ± 0.09 (*n* = 8 per group, mean ± SD). (B) RT-qPCR was performed to detect the impact of serum from different groups on the expression level of MICU3 pre-mRNA. Peripheral blood-Neutrophil: Sham: 1.00 ± 0.10, MIRI: 3.80 ± 0.42; Neutrophil-like-HL60: Sham: 1.00 ± 0.11, MIRI: 5.39 ± 0.57 (*n* = 8 per group, mean ± SD). (C) The lactate levels in serum samples from the MIRI group and the Sham group were measured. Sham: 169.65 ± 14.76, MIRI: 293.37 ± 20.12 (*n* = 20 filed per group, mean ± SD). (D) Schematic diagram depicting HL60 cells and peripheral blood neutrophil treated with or without lactate. (E) The effects of lactate on NET activation levels were assessed by IF assay (*n* = 20). (F) RT-qPCR was utilized to examine the impact of increasing concentrations of lactate on the levels of MICU3 pre-mRNA and mRNA. Neutrophil-like-HL60: pre-MICU3: Sham: 1.00 ± 0.11, MIRI: 1.40 ± 0.17; mRNA: Sham: 3.10 ± 0.35, MIRI: 3.90 ± 0.45; Peripheral blood-Neutrophil: pre-MICU3: Sham: 1.00 ± 0.10, MIRI: 1.80 ± 0.23; mRNA: Sham: 3.70 ± 0.40, MIRI: 5.82 ± 0.63; Neutrophil-like-HL60: MICU3: Sham: 1.00 ± 0.12, MIRI: 2.48 ± 0.28; mRNA: Sham: 5.07 ± 0.54, MIRI: 7.77 ± 0.83; Peripheral blood-Neutrophil: MICU3: Sham: 1.00 ± 0.11, MIRI: 2.08 ± 0.22; mRNA: Sham: 5.08 ± 0.52, MIRI: 6.35 ± 0.64 (*n* = 8 per group, mean ± SD).

To investigate the mechanism behind the up-regulation of MICU3 in MIRI, we treated HL60 cells with serum derived from both the Sham and MIRI groups. We then assessed the activity of the MICU3 promoter region through luciferase reporter assay. The findings revealed that the addition of serum from the MIRI group did not enhance the activity of the MICU3 promoter (Fig. 5A). However, RT-qPCR results indicated that serum from the MIRI could up-regulate MICU3 pre-mRNA expression (Fig. 5B), suggesting that serum derived from the MIRI group can activate the transcription of the MICU3 gene without affecting its promoter activity. Lactate levels were significantly elevated under ischemic/hypoxic conditions [20], and lactate has been reported to promote gene transcription by influencing histone lactylation modifications [21,22]. Based on this background, we hypothesized that the transcription of MICU3 may be mediated by lactate-induced histone lactylation. We measured the lactate levels in serum samples from both the MIRI and Sham groups, finding that the lactate concentration was significantly higher in the MIRI group than in the Sham group (Fig. 5C). This suggests that lactate may play a role in mediating the high expression of MICU3. We added lactate to neutrophils and HL60 cells treated with serum from the Sham group (Fig. 5D). IF results demonstrated an increase in NET activation in response to lactate treatment (Fig. 5E), indicating that lactate promotes NET activation. Subsequently, we treated neutrophils and HL60 cells with different concentrations of lactate and used RT-qPCR to detect the levels of MICU3 mRNA and pre-mRNA to analyze the influence of lactate on MICU3 expression, revealing that the activation effect of lactate on MICU3 expression was dose-dependent (Fig. 5F).

**Fig. 6.**
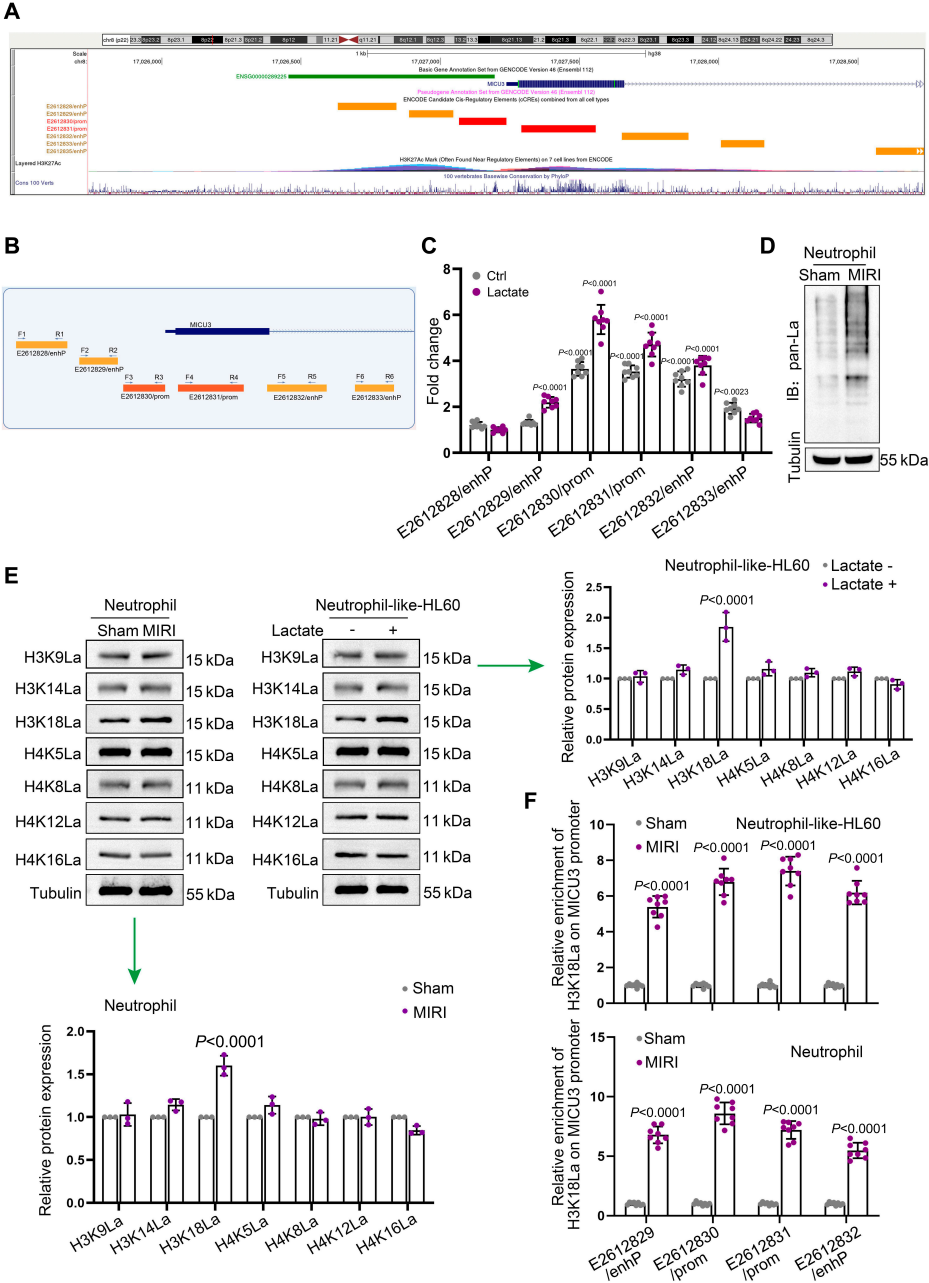
Lactate up-regulates MICU3 expression by mediating H3K18la. (A) The TSS of the MICU3 gene, along with upstream and downstream promoter and enhancer signal sites, was obtained from the UCSC website. (B) A schematic diagram was created for primer design. (C) ATAC-qPCR was performed to assess chromatin accessibility at potential regulatory sites within the MICU3 gene promoter region before and after lactate treatment. (D) WB was used to detect changes in the total levels of total lactylation modifications in neutrophils from the Sham and MIRI groups (*n* = 3 per group). (E) WB was conducted to analyze the changes in lactylation modification levels at various histone sites in neutrophils and HL60 cells from different groups (*n* = 3 per group). (F) ChIP was performed to verify the increased H3K18 lactylation modification at the promoter and enhancer of the MICU3 gene in the Sham and MIRI groups. Neutrophil-like-HL60: E2612829/enhP: Sham: 1.00 ± 0.10, MIRI: 5.40 ± 0.60; E2612830/prom: Sham: 1.01 ± 0.11, MIRI: 6.80 ± 0.74; E2612831/prom: Sham: 1.01 ± 0.12, MIRI: 7.40 ± 0.80; E2612832/enhP: Sham: 1.00 ± 0.09, MIRI: 6.19 ± 0.66. Neutrophil: E2612829/enhP: Sham: 1.00 ± 0.11, MIRI: 6.79 ± 0.71; E2612830/prom: Sham: 1.01 ± 0.13, MIRI: 8.60 ± 0.92; E2612831/prom: Sham: 1.00 ± 0.10, MIRI: 7.20 ± 0.75; E2612832/enhP: Sham: 1.01 ± 0.11, MIRI: 5.48 ± 0.65 (*n* = 8 per group, mean ± SD).

By applying the UCSC (University of California, Santa Cruz Genomic Browser Database) website, we obtained the promoter and enhancer signal sites upstream and downstream of the transcription start site (TSS) of the MICU3 gene and designed corresponding primers (Fig. 6A and B). We then performed ATAC (assay for transposase accessible chromatin) to obtain DNA products from open chromatin regions in both the control and lactate-treated groups, assessing the enrichment levels of each signal site through qPCR (Fig. 6C), which indicated that under lactate treatment, the chromatin at the promoter and enhancer sites of MICU3 was opened, suggesting that the MICU3 gene is activated in response to lactate. We further assessed the levels of lactylation modifications in neutrophils from different groups using WB and found that the overall level of lactylation modifications increased (Fig. 6D).

Additionally, we investigated the alterations in lactylation modification levels at histone sites associated with enhanced gene expression, such as H3K9, H3K14, H3K18, H4K5, H4K8, H4K12, and H4K16. Notably, H3K18la exhibited a significant increase (Fig. 6E). Finally, we conducted chromatin immunoprecipitation in neutrophils from both the Sham and MIRI groups. The results showed an increase level of H3K18la modification level at the promoter and enhancer regions of the MICU3 gene in the MIRI group (Fig. 6F), indicating that lactate-mediated H3K18la promotes MICU3 transcription. Besides, as is well known, acetylation modification at the H3K27 site of H3 promotes gene transcription activity [23]. To ascertain whether H3K27Ac contributes to the lactate-induced high expression of MICU3, we performed ChIP experiments to obtain DNA fragments with acetylation modification at the H3K27 site and assessed the enrichment level in the MICU3 gene promoter region in HL60 cells and neutrophils treated with or without lactate. The results showed no significant difference in the H3K27ac modification level in the MICU3 gene promoter region under lactate treatment (Fig. S3A), indicating that H3K27Ac modification does not contribute to the lactate-induced expression of MICU3.

### Lactate/AARS1 enhances the stability of MICU3 protein through lactylation modification, promoting NET activation

**Fig. 7.**
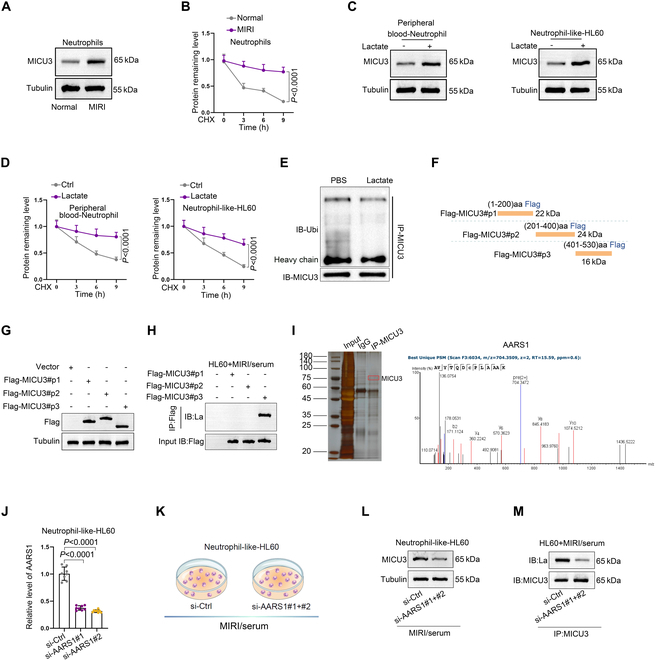
Lactate/AARS1 enhances the stability of MICU3 protein through lactylation modification, promoting NET activation. (A) WB assay was performed to detect the level of MICU3 in clinical samples (*n* = 3). (B) Neutrophils were treated with CHX, and WB assay was performed to determine the level of MICU3 (*n* = 3). (C) Under the influence of lactate, WB was used to detect the expression of MICU3 protein in HL60 cells and neutrophils derived from peripheral blood (*n* = 3). (D) Under the influence of lactate, the CHX chase assay was conducted to assess the stability of MICU3 protein (*n* = 3). (E) WB was used to determine the ubiquitination modification level of MICU3 under lactate treatment (*n* = 3). (F) Fusion proteins with Flag tags were constructed for different MICU3 protein segments: P1 (1–200), P2 (201–400), and P3 (401–530). (G) After transfecting HL60 cells with the fusion expression proteins, WB was utilized to verify the expression efficiency (*n* = 3). (H) Co-IP was conducted to detect differences in lactylation modification levels among the 3 segments (*n* = 3). (I) Analysis of silver staining and MS results of binding proteins of MICU3 obtained from IP experiments (*n* = 3). (J) RT-qPCR was used to validate the knockdown efficiency of AARS1 in HL60 cells. si-Ctrl: 1.01 ± 0.12, si-AARS1#1: 0.37 ± 0.04, si-AARS1#2: 0.32 ± 0.03 (*n* = 8 per group, mean ± SD). (K to M) Changes in the lactylation modification levels of MICU3 and the expression of MICU3 protein were assessed after knockdown of AARS1 (*n* = 3).

Lactylation modifications not only regulate gene transcription by modulating histone lactylation but also can mediate protein modifications that enhance protein stability or activity [24–26]. However, the effect of lactate on MICU3 protein in the context of MIRI has yet to be fully explored. We first examined the levels of MICU3 in neutrophils from healthy individuals and MIRI patients in peripheral blood, and also analyzed the degradation of MICU3 after treatment with the protein synthesis inhibitor cycloheximide (CHX). The results showed that MICU3 levels in neutrophils from MIRI patients were significantly higher than those in the control group, and its stability was also significantly higher than that in the control group (Fig. 7A and B). We added lactate to neutrophils or HL60 cells and performed WB analysis to assess the protein levels of MICU3. We found that the MICU3 protein level increased (Fig. 7C). Subsequent experiment using CHX indicated that lactate could enhance the stability of the MICU3 protein (Fig. 7D), suggesting that lactate may influence MICU3 protein stability through lactylation modification. Studies have reported that protein lactylation can inhibit protein ubiquitination degradation [27]. Through protein IP experiments, we analyzed the impact of lactate on the ubiquitination modification of the MICU3 protein. To eliminate the influence of MICU3 protein expression levels, we deliberately standardized the MICU3 content. The WB results confirmed a decrease in the ubiquitination modification level of MICU3 under lactate treatment (Fig. 7E).

MICU3 consists of 530 amino acids, and we first predicted potential lactylation modification sites on the MICU3 amino acid sequence using the online tool DeepKla (Fig. S3B), identifying 7 sites with a probability greater than 1%. To further analyze the lactylation modification sites on MICU3, we constructed Flag-tagged fusion proteins of MICU3 with amino acid sequences corresponding to predicted domain distributions: P1 (1–200), P2 (201–400), and P3 (401–530) (Fig. 7F). We transfected the fusion vector into HL60 cells, and we verified the expression efficiency through WB analysis (Fig. 7G). We performed IP experiments in MIRI-derived serum from the MIRI-treated HL60 cells transfected with indicated vectors, followed by WB analysis to detect the lactylation modification levels. The results showed that only the P3 segment exhibited enriched lactylation modifications (Fig. 7H), indicating that lactylation occurs at the P3 segment of MICU3. Furthermore, we performed MS analysis on the proteins that bound to MICU3 obtained from the IP experiments and identified AARS1 as a binding protein (Fig. 7I).

Previous studies have reported that AARS1 mediates lactylation modifications of proteins [24,25]. Thus, we hypothesized that AARS1 mediates the lactylation of MICU3, enhancing its protein stability. To test this hypothesis, we knocked down AARS1 in HL60 cells (Fig. 7J) and performed WB analysis, which revealed a decrease in both the lactylation modification level of MICU3 and MICU3 protein expression (Fig. 7K to M). Additionally, we found that knocking down AARS1 reduced the activation level of NETs (Fig. S3C) and decreased the intracellular Ca^2+^ levels in HL60 cells (Fig. S3D). We are aware that another member of the AARS family, AARS2, is primarily localized in the mitochondria. In order to analyze whether AARS2 might also be involved in the regulation of MICU3, we examined whether the colocalization of AARS1 and AARS2 with MICU3 is affected by lactic acid. The results show that the colocalization of AARS1 with MICU3 is more significantly affected by lactic acid (Fig. S3E), indicating that although AARS2 is localized in the mitochondria, it does not participate in lactic acid-mediated regulation of MICU3. These results indicate that MICU3 undergoes lactylation modification under the influence of AARS1, enhancing its protein stability and promoting NET activation.

### Animal-level validation shows that NET generation exacerbates microvascular endothelial cell injury and worsens MIRI

**Fig. 8.**
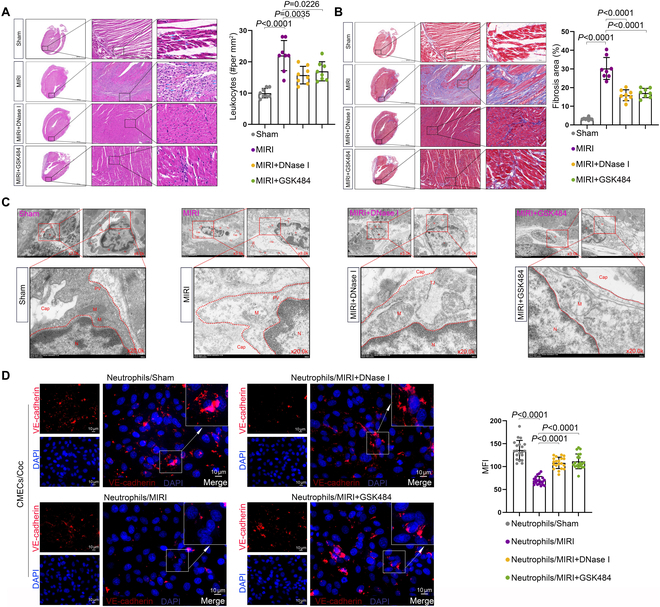
Animal-level validation shows that NET generation exacerbates microvascular endothelial cell injury and worsens MIRI. (A) H&E staining was used to assess inflammatory infiltration in tissue samples from different groups. Sham: 10.00 ± 1.51, MIRI: 22.00 ± 4.81, MIRI + DNase I: 15.75 ± 2.82, MIRI + GSK484: 16.97 ± 3.06 (*n* = 8 per group, mean ± SD). (B) Masson staining was conducted to detect fibrosis in tissue samples from different groups. Sham: 3.29% ± 0.64%, MIRI: 30.28% ± 5.87%, MIRI + DNase I: 15.91% ± 2.92%, MIRI + GSK484: 17.12% ± 2.48% (*n* = 8 per group, mean ± SD). (C) TEM was used to observe changes in the microvascular structure of myocardial tissue. Cap, vascular lumen; Enc, endothelial cells; N, nucleus; M, mitochondria; PV, pinocytotic vesicles; TJ, tight junctions (*n* = 3). (D) IF was performed to detect the expression of VE-cadherin of HUVECs under different treatments. Neutrophils/Sham: 136.08 ± 20.98, Neutrophils/MIRI: 69.45 ± 8.91, Neutrophils/MIRI + DNase I: 108.14 ± 12.38, Neutrophils/MIRI + GSK484: 111.44 ± 16.16 (*n* = 20 filed per group, mean ± SD).

In addition to establishing Sham and MIRI rat models, we also created a NET inhibition model [deoxyribonuclease (DNase) I used to degrade NETs and GSK484 used to inhibit PAD4]. Histological analysis using H&E staining and Masson staining showed that the addition of NET inhibitors alleviated inflammatory infiltration and fibrosis during the MIRI process (Fig. 8A and B). Furthermore, TEM observations revealed that the extent of endothelial structural damage was severe in the MIRI group, while inhibition of NETs by DNase I resulted in reduced endothelial structural damage (Fig. 8C). Furthermore, we cocultured neutrophils from different groups with endothelial cells to analyze the expression of VE-cadherin in endothelial cells as a measure of endothelial damage. The results showed that neutrophils from MIRI patients significantly damaged the endothelium, and inhibiting NET formation could reverse this phenomenon (Fig. 8D). These experimental results confirm that in vivo, the generation of NETs by neutrophils exacerbates microvascular endothelial cell injury, thereby worsening MIRI.

## Discussion

IHD is a major global leading cause of death, with its pathophysiology being multifactorial, involving multiple factors such as inflammation, microvascular dysfunction, and oxidative stress. Although reperfusion therapies like PCI can effectively restore blood supply to the myocardium, their potential side effects, such as MIRI, pose challenges to the application of these treatment strategies. This study specifically focuses on the role of NETs in MIRI and investigates the mechanisms behind their activation.

The regulatory mechanisms of MIRI involve various factors, such as transcription factors, miRNAs, circular RNAs [28], and so on. Specifically, FOXN4 modulates MIRI through ferroptosis mediated by the hypoxia-inducible factor-1α (HIF-1α)/matrix metalloproteinase 2 (MMP2) pathway [29]; miR-190-5p reduces cell apoptosis levels by targeting PHLPP1, improving MIRI [30]; circMIRIAF sponges miR-544 to up-regulate WDR12 expression, thereby exacerbating MIRI [31]; and exercise promotes cardiomyocyte proliferation and survival by inducing miR-210 to target DK10 and EFNA3, thereby effectively alleviating MIRI [32]. Additionally, immune cells also play an important role in MIRI. For example, interleukin-38 (IL-38) alleviates MIRI by inhibiting the activation of inflammatory macrophages [33]. However, our research specifically aims to elucidate the mechanisms by which neutrophils contribute to MIRI.

NETs are net-like structures composed of DNA, histones, and antimicrobial proteins released by activated neutrophils in response to various stimuli. The formation and activation of NETs have dual effects; on one hand, they effectively control bacterial infections, while on the other hand, excessive NET formation can lead to vascular occlusion, resulting in further damage and inflammation [34,35]. Previous research has shown a strong link between NETs and a range of diseases, including tumors [36], fibrotic diseases [37], chronic/acute inflammation [38], ocular diseases [39], and diabetes [40].

In this study, we constructed a MIRI model in rats, and through echocardiography, ELISA, H&E staining, Masson staining, and electron microscopy, we observed that the MIRI process was accompanied by CMEC injury and inflammatory infiltration. Previous studies have indicated that NETs can promote cerebral ischemia/reperfusion injury (CI/RI) [41] and lung ischemia–reperfusion injury (IRI) [42]. We detected high level of NETs in the myocardial tissue and peripheral blood of rats in the MIRI group. Furthermore, we found that NETs from the MIRI group could induce apoptosis and inhibit tube formation of CMECs/HUVECs. These findings align with previous studies suggesting that NETs can directly damage parenchymal cells in other ischemia–reperfusion contexts [43].

NETs are regulated by various mechanisms. For instance, PAD4 expression positively regulates NETs in severe asthma [44], and CEACAM1 influences NET formation by regulating the S1P-S1PR2/S1PR3 axis [45]. However, this study reveals a novel activation mechanism of NETs in MIRI. We found that the mitochondrial Ca^2+^ uptake-related gene MICU3 was significantly elevated in MIRI. Subsequently, we confirmed that MICU3 promotes mitochondrial Ca^2+^ uptake, mitophagy, and NET formation in neutrophils. To further investigate the regulatory mechanism of MICU3 on NET formation, we performed IP-MS to identify the binding protein VDAC1. Our experiments demonstrated that the interaction between MICU3 and VDAC1 facilitates mitochondrial Ca^2+^ uptake, causing mitochondrial dysfunction that leads to mitophagy and NET generation. Previous literature has indicated that excessive mitochondrial Ca^2+^ accumulation plays a critical role in hepatic ischemia-reperfusion injury [46], and our results support this, indicating that mitochondrial Ca^2+^ overload plays a similar role in MIRI.

Additionally, we explored the upstream mechanisms that regulate MICU3 expression. By measuring lactate levels in the serum of different groups of rats, we found that the lactate concentration was significantly elevated in the MIRI group. Previous literature has reported that lactate can mediate H3K18lac, promoting gene transcription [47]. Consistent with this, our study indicates that lactate can enhance MICU3 transcription through H3K18lac. Furthermore, we discovered that AARS1 binds to MICU3, catalyzing its lactylation modification, which in turn enhances its protein stability.

Although we have conducted this work, our study still has some shortcomings. For instance, we mainly focused on myocardial microvascular endothelial cells, without examining the impact of NETs on myocardial cells themselves. Despite existing studies confirming that NETs can harm myocardial cells [43], this remains a limitation in our research. In future studies, we will systematically investigate the overall effects of NETs on myocardial cells and CMECs, as well as the connections between them, to further elucidate the mechanisms by which NETs cause damage in MIRI.

Finally, in summary, our study reveals that MICU3-mediated mitochondrial calcium uptake, mitophagy, and the subsequent activation of NETs play a key role in exacerbating MIRI. NETs contribute not only to microvascular dysfunction but also directly to myocardial cell injury, providing a more comprehensive understanding of their pathological role in MIRI. Additionally, we identify lactate as a potential upstream regulator of MICU3 expression, offering novel insights into the molecular mechanisms that contribute to the pathogenesis of MIRI. These findings open new therapeutic avenues for targeting NETs and mitochondrial dysfunction in IHD and related cardiovascular diseases.

## Conclusion

This study reveals that lactate enhances MICU3 transcription and protein stability, promoting its interaction with VDAC1 to form MAMs. This triggers excessive mitochondrial Ca^2+^ uptake, mitochondrial dysfunction, and mitophagy and ultimately activates NETs, leading to microvascular endothelial cell damage and aggravating MIRI. These findings offer new therapeutic insights for MIRI and IHD.

## Materials and Methods

### Establishment of the MIRI model in rats

specific pathogen-free (SPF)-grade male Sprague–Dawley rats, 8 weeks old and weighing 280 to 300 g, were applied. To establish the MIRI model, the rats were first weighed, and preoperative electrocardiograms were performed. The rats were then connected to a ventilator and anesthetized with isoflurane using a small animal anesthesia machine (RWD, Shenzhen, China). The MIRI surgery involved occluding the left anterior descending coronary artery by directly ligating it with a vascular clamp 3 mm from the root of the aorta, resulting in complete myocardial ischemia. After 30 min of occlusion, the vascular clamp was removed to restore myocardial reperfusion. In the Sham surgery group, after thoracotomy, a suture was placed around the left anterior descending coronary artery without ligation. For NET inhibitor-treated group, rats were intracardiac injected with DNase I (MCE, Monmouth Junction, NJ, USA) [48] 2.5 mg/kg once a day for 7 consecutive days. After 7 d, the rats were harvested for subsequent experiments.

### Echocardiography

During the ischemia–reperfusion period, echocardiographic assessments were conducted at 4 time points: prior to ischemia, after 15 min of ischemia, after 15 min of reperfusion, and on postoperative day 7. Cardiac function in each group was assessed using a high-resolution small animal ultrasound system (Vevo LAZR-X, Fujifilm Visualsonics) equipped with an MX250 probe. After anesthetizing the rats, they were positioned supine with their limbs and head secured, followed by shaving the chest area. In the short-axis view of the left ventricle, M-mode was used to record left ventricular motion, and the heart rate and other indicators were measured. Finally, left ventricular ejection fraction (LVEF) and left ventricular fractional shortening (LVFS) were subsequently calculated.

### H&E staining and Masson staining

Seven days after ischemia–reperfusion and following DNase I treatment, rats from each group were randomly selected for euthanasia to collect cardiac tissue for H&E staining and Masson staining experiments.

### Determination of myocardial enzyme spectrum

Twenty-four hours after ischemia–reperfusion, the levels of CK-MB, LDH, and cTnI in the serum were measured using a semi-automated biochemical analyzer.

### Preparation of serum

Twenty-four hours after ischemia–reperfusion, the rats from each group were anesthetized and positioned supine. After exposing the abdominal aorta and performing routine disinfection, an incision was made along the abdominal midline to locate the abdominal aorta. A puncture needle was inserted into the abdominal aorta at an angle of less than 30°, and blood was collected using EDTA anticoagulant tubes. The collected blood was promptly processed for serum separation, with centrifugation conditions set as 3,000 rpm at room temperature for 10 min.

### Extraction of neutrophils from rat peripheral blood

Neutrophils were extracted using the rat peripheral blood neutrophil separation kit (Solarbio, P9200), following the manufacturer’s instructions. As previously described [49], a second centrifugation was performed using a Percoll gradient of 55%, 65%, 70%, and 80% to isolate neutrophils. The 70% Percoll layer, including the boundary cell layer, was collected and washed with Hanks’ balanced salt solution. Subsequently, the neutrophils were incubated with 1% serum from different groups for 3 h before proceeding to the next experimental steps. The supernatant of neutrophil culture was centrifuged at 4 °C, 18,000*g* for 10 min to collect the precipitate, which is referred to as NETs. After resuspending in phosphate-buffered saline (PBS), the MPO level was assessed using ELISA to evaluate the NET level.

### Extraction of neutrophils from human peripheral blood

Peripheral blood samples were collected from 48 healthy individuals undergoing physical examinations and 48 patients with MIRI from Pukou Hospital of Chinese Medicine affiliated to China Pharmaceutical University. All studies obtained informed consent from the participants and approval from the ethics review board. Neutrophils were isolated using neutrophil separation solution (e.g., Solarbio, P9402) according to the manufacturer’s instructions. The extraction process involved mixing blood, saline, and red blood cell sedimentation fluid in a 1:1:1 ratio, at room temperature for 35 min, and carefully transferring the supernatant (rich in white blood cells and platelets) for subsequent separation. The supernatant was supplemented with reagent A to a total volume of 4 ml, and then 2 ml of reagent C was gently layered on top of reagent A. The mixture was centrifuged at 800*g* for 30 min at room temperature, and the lower circular milky white layer, representing the desired neutrophil layer, was collected. The cells were washed with 10 ml of cell washing solution at 250*g* for 10 min. The supernatant was discarded, and the cells were resuspended in 5 ml of cell washing solution, centrifuged at 250*g* for 5 min. The supernatant was discarded, and the cells were resuspended for further use.

### Rat CMEC extraction

Rats were anesthetized and fixed in a supine position. After disinfection, the chest cavity was opened layer by layer to retrieve the heart. The heart was then rinsed for 3 to 5 min using a pre-cooled, oxygen-saturated perfusion buffer. The separation of cells was performed according to previously described methods [50]. The isolated cells were cultured in complete Dulbecco’s modified Eagle’s medium containing 1× Glutamax, 1 ng/ml basic fibroblast growth factor (MCE, HY-P5321), and 10% fetal bovine serum (BI, C04001-0500).

### Cell culture

HL60 cells were obtained from iCell Bioscience Inc. (Shanghai, China) and cultured using HL60-specific culture medium (iCell-h098-001b). HL-60 cells were differentiated into ​mature neutrophils by treatment with ​1.25% DMSO and 1 μM all-trans retinoic acid (ATRA) before experimentation. HUVECs were also sourced from iCell Bioscience Inc. and cultured using HUVEC-specific culture medium (iCell-h110-001b). CQ (MCE, HY-17589A) was used to treat the cells at a working concentration of 20 μM for 24 h, with the control group receiving an equal volume of dimethyl sulfoxide. Lactate (MCE, HY-B2227) was applied at working concentrations of 5, 10, and 20 mM for 24 h, with the control group receiving an equal volume of saline solution.

### Cell transfection

In a 6-well plate, 2 ml of HL60 cells at a density of 4 × 10^5^ cells/ml was seeded into each well, resulting in a total cell count of 8 × 10^5^ cells. For transfection, 6 μg of Vector/OE-MICU3 plasmid was mixed directly with 6 μl of Advanced DNA RNA Transfection Reagent (Zeta Life, AD600025), or 6 μl of 20 μM small interfering RNA (siRNA) was mixed with 6 μl of Advanced DNA RNA Transfection Reagent. The mixture was carefully pipetted 10 to 15 times to achieve homogeneity and then incubated at room temperature for 15 min prior to being added to the cells.

### IF assay

Following deparaffinization and sequential rehydration of tissue paraffin sections, the endogenous peroxidase activity was inhibited. Antigen retrieval was carried out with Tris-EDTA buffer at a pH of 9.0. Following permeabilization, antigen blocking was conducted using 10% goat serum. Subsequently, primary antibodies were incubated at 4 °C for 2 h to overnight. Then, secondary antibodies were applied at room temperature for 1 to 2 h. Following incubation, the nuclei were stained using Hoechst 33342 fluorescent dye (Beyotime, C1029) and incubated at room temperature for 15 min before washing. The antifade mounting medium (Beyotime, P0126-5ml) was applied to the tissue, and coverslips were mounted.

For the cell adhesion slide samples, after fixation, permeabilization, and blocking, primary antibody incubation was performed according to the recommended dilution ratio. Corresponding secondary antibodies were chosen based on the primary antibodies, followed by incubation and washing. Finally, the slides were inverted onto coverslips containing the antifade mounting medium.

### Tube formation assay

The tube formation assay will be performed using RGF BME, Type R1 (R&D Systems, 3433-001-R1). After seeding the cells for 6 h, tube formation capability was quantified using ImageJ, with 5 random areas selected for photography from each group. The length of the tubes was calculated by drawing a line along each tube, and the length of this line was measured in micrometers using ImageJ with a scale. The average of 3 fields of view was taken as the final result.

### Flow cytometry for apoptosis

Endothelial cells treated according to the experimental groups were digested using trypsin (Beyotime, Shanghai, China, C0205) and collected. The cells will be stained using the flow cytometric apoptosis detection kit (Beyotime, C1062S) and analyzed using a flow cytometer.

### Mitochondrial calcium ion labeling

Mitochondrial calcium ions were detected using the ​calcein AM fluorescent probe combined with ​cobalt chloride (CoCl₂) quenching. Cells were labeled with ​2 μM calcein AM (Beyotime, C2012-01) and ​80 mM CoCl₂ at ​37 °C in the dark for 15 min. After staining, cells were washed with ​PBS, resuspended in PBS, and analyzed by ​flow cytometry.

### CHX chase assays

Cells were seeded into 6-well plates according to the experimental groups, with 100 μM CHX (MCE, HY-12320) added to each well. The cells were treated for 0, 3, 6, and 9 h. After treatment, the cells were collected for further analysis.

### TEM and SEM

After anesthetizing the rats, they were perfused sequentially with pre-cooled PBS, 0.25% glutaraldehyde at 37 °C, and pre-cooled 0.25% glutaraldehyde. Subsequently, secondary fixation was performed. After staining, images were collected using a transmission electron microscope (Hitachi, HT7800) at an acceleration voltage of 80 kV. The samples for SEM were observed and imaged using a scanning electron microscope (Hitachi, SU8100).

### RT-qPCR

RNA extraction was performed using Trizol (Invitrogen, 15596026CN). Reverse transcription was conducted with a reverse transcription kit (Vazyme, R312), and gene expression levels were assessed using a qPCR kit (Vazyme, Q312). The experimental procedures and instrument settings were carried out according to the product manuals. Reverse transcription experiments were completed using a gradient PCR instrument (Bio-Rad, T100), and the real-time fluorescence quantitative PCR melting curve program was operated using a qPCR instrument (HealForce, CG-05) with the built-in program.

### Western blot

Fifty milligrams of tissue or cells was taken to extract proteins. The proteins were separated by size using sodium dodecyl sulfate–polyacrylamide gel electrophoresis gel, followed by transfer to a membrane and blocking. After blocking, the primary antibodies were added and incubated for 1 to 2 h, and then the secondary antibodies were added and incubated for another 1 to 2 h, with washing performed using TBST in between. The membrane, soaked in enhanced chemiluminescence solution, was placed in a chemiluminescence imaging system for band development. Detection of histone lactylation modifications was performed using the Lactyl-Histone Antibody Sampler Kit (PTM bio, PTM-7093).

### Co-immunoprecipitation

Co-IP for MICU3 (Bioss, Woburn, MA, USA, bs-14517R) and Flag (Proteintech, 20543-1-AP) was conducted in treated HL60 cells. Cells were first lysed using IP Lysis Buffer (Pierce, 87787), with 1 mg of protein taken from each group for the experiment. Antibodies were used at a concentration of 3 μg, and rabbit immunoglobulin G served as the negative control. Protein A/G Magnetic beads (MCE, HY-K0202) were utilized to capture the immunoprecipitated protein complexes. The magnetic bead–protein complexes were washed with IP Wash Solution and PBS buffer. The enrichment of captured protein complexes was assessed using WB or silver staining.

### Mitochondrial and ER staining

The MitoTracker Red (Thermo Fisher, Waltham, MA, USA, A66443) dye was employed for mitochondrial labeling in neutrophils, while the ER-Tracker Green (Thermo Fisher, E34251) dye was used for ER labeling in neutrophils. Images were captured and recorded using a fluorescence microscope.

### Perform PLA

Cell or tissue slices were fixed, permeabilized, and incubated with specific primary antibodies (VDAC1, Proteintech, 66345-1-Ig; MICU3, Thermo Fisher, PA5-55177) against the target protein after blocking; 2 PLA secondary antibodies with unique DNA sequences (such as plus/minus probes) that bind to the primary antibody were introduced; when the 2 probes are within <40 nm, DNA ligase circularizes their complementary sequences, generating repeated DNA products through rolling circle amplification; fluorescently labeled complementary probes were added, and punctate signals were observed under a fluorescence microscope, where each dot represents a single protein interaction or modification event.

### Lactate content measurement

The serum lactate content was measured using the Lactic Acid (L-LA) Content Assay Kit (colorimetric method) (Sangon, Shanghai, China, D799851-0050), following the protocol provided in the kit instructions. A standard curve was established, and absorbance was measured at 570 nm.

### ChIP-qPCR

A final concentration of 1% high-concentration paraformaldehyde was added to the cells for crosslinking for 15 min, and the crosslinking was terminated with glycine solution. After washing with PBS, nuclear-cytoplasmic separation was performed to obtain the nuclei. Chromatin was fragmented into 100- to 1,000-base pair pieces using a sonicator (SCIENTZ, JY92-IIN). After diluting the chromatin fragments, H3K18la antibody was added, and the mixture was rotated overnight at 4 °C. Protein A/G magnetic beads (MCE, HY-K0202) were then added, and the mixture was rotated for an additional 2 h at 4 °C. The washing buffer was used to sequentially wash the magnetic beads, with gentle vortexing during each wash. After allowing the magnetic stand (Sangon, D601021) to adsorb for 1 min, the supernatant was removed. Following the final wash, crosslink reversal was performed, and DNA purification was carried out using a DNA purification kit (Vazyme, DC301-01).

### ATAC-qPCR

The experiment was conducted as described by a previous study [51]. The fragmented products were purified using a DNA purification kit (Vazyme, DC301-01), and elution was performed with 20 μl of elution buffer. The eluted DNA products were analyzed for chromatin accessibility using a qPCR kit (Vazyme).

### Statistical analysis

All experiments performed in this study were repeated a minimum of 3 times. Data analysis was carried out using SPSS version 24.0, and results were expressed as mean ± standard deviation (SD) with GraphPad Prism version 8.0.2. Statistical significance between groups was established with a *P* value of less than 0.05. Student’s *t* test was used for comparisons between 2 samples, while one-way analysis of variance (ANOVA) followed by Tukey’s post hoc test was utilized for comparisons involving more than 2 samples.

## Data Availability

All data generated or analyzed during this study are included in this article. Further enquiries can be directed to the corresponding author.
