# Supplementary material for: Lactate-Induced Mitochondrial Calcium Uptake 3 Aggravates Myocardial Ischemia–Reperfusion Injury by Promoting Neutrophil Extracellular Trap Formation
Source: Research (Wash D C). 2025 May 30;8:0705. doi: 10.34133/research.0705 (PMC12123085; doi:10.34133/research.0705)
Supplement: Supplementary 1 — Figs. S1 to S3 [file research.0705.f1.zip › Supplementary Information.docx]

**Supplementary Information**

**Figure S1.**

**A.** Quantitative analysis of tube formation assays to analyze the angiogenic potential of HUVECs (n=15).

**B.** Flow cytometry was used to analyze apoptosis rate of HUVECs. Sham: 5.88±0.67, MIRI: 15.43±1.94(n=8 per group, mean±SD).

**C.** IF was performed to detect the expression of VE-cadherin in HUVECs under different treatments. Sham: 137.97±15.53, MIRI: 63.61±8.95 (n=20 per group, mean±SD).

**D**. Flow cytometry was conducted to detect differences in intracellular Ca^2+^ levels of HL60 cells from different treatment groups. Sham/serum: Vector: 12.06±1.78, OE/MICU3: 27.59±2.91; MIRI/serum: siCtrl:13.43±1.98, si-MICU3#1+2: 3.55±0.30 (n=8 per group, mean±SD).

**E.** Mitophagy inhibitors were added to treat endogenous neutrophils (derived from MIRI rats) and neutrophil-like cells (transfected with MICU3 overexpression vectors), both of which were treated with MIRI serum.

**F.** WB was used to detect differences in the expression of mitophagy-related proteins among different treatment groups (n=3).

**G.** Quantitative analysis of IF to assess differences in NETs activation levels among cells from different treatment groups. Model#1: No CQ: 21.99±3.04, With CQ: 9.00±1.05; Model#2: No CQ: 28.98±3.75, With CQ: 14.00±1.77 (n=20 per group, mean±SD).

**Figure S2.**

**A.** Mito-Tracker and ER-Tracker staining was conducted to detect level of MAMs in different groups (n=20).

**B.** RT-qPCR was performed to validate the knockdown efficiency of VDAC1 in HL60 cells. si-Ctrl: 1.01±0.10, si-VDAC1#1: 0.37±0.04, si-VDAC1#2: 0.47±0.45 (n=8 per group, mean±SD).

**C.** The Ca^2+^ content in HL60 cells with MICU3/VDAC1 knockdown, treated with MIRI serum was assessed. si-Ctrl: 13.66±1.60, si-VDAC1#1+2: 2.32±0.21, si-MICU3#1+2: 4.28±0.60 (n=8 per group, mean±SD).

**D.** WB was conducted to detect the levels of mitophagy-related proteins in MICU3/VDAC1 knockdown HL60 cells treated with MIRI serum (n=3).

**E.** The levels of NETs in HL60 cells with knockdown of MICU3/VDAC1 treated with MIRI serum was assessed. si-Ctrl: 35.01±3.76, si-VDAC1#1+2: 12.00±1.70, si-MICU3#1+2: 15.00±2.18 (n=8 per group, mean±SD).

**Figure S3.**

**A.** ChIP-qPCR was used to determine the H3K27ac modification level in the MICU3 gene promoter region under lactate treatment. HL-60: Ctrl (IgG: 1.00±0.12, H3K27ac: 11.91±1.22), Lactate (IgG: 1.00±0.14, H3K27ac: 12.90±1.89); Peripheral blood-Neutrophil: Ctrl (IgG: 1.00±0.13, H3K27ac: 8.13±1.01), Lactate (IgG: 1.00±0.10, H3K27ac: 7.45±0.79) (n=8 per group, mean±SD).

**B.** Potential lactylation modification sites on the MICU3 amino acid sequence were predicted using the online tool DeepKla (http://lin-group.cn/server/DeepKla/index.html).

**C-D.** Knockdown of AARS1 was found to reduce NETs activation levels and Ca^2+^ levels in HL60 cells (n=20).

**E.** IF assay was utilized to detected the co-localization between MICU3 with AASR1 or AASR2 (n=20).
