# Supplementary figures and images for: Lactate-Induced Mitochondrial Calcium Uptake 3 Aggravates Myocardial Ischemia–Reperfusion Injury by Promoting Neutrophil Extracellular Trap Formation
Source: Research (Wash D C). 2025 May 30;8:0705. doi: 10.34133/research.0705 (PMC12123085; doi:10.34133/research.0705)

A

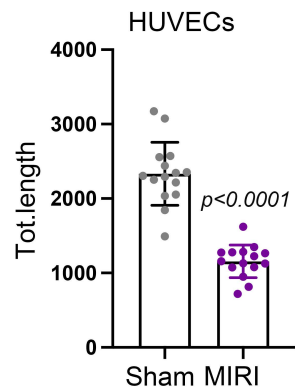

C

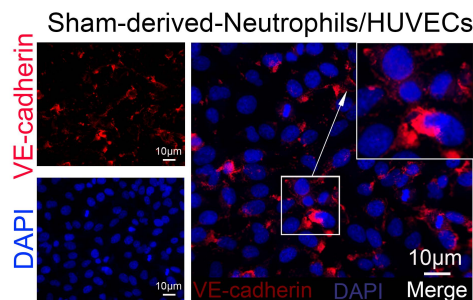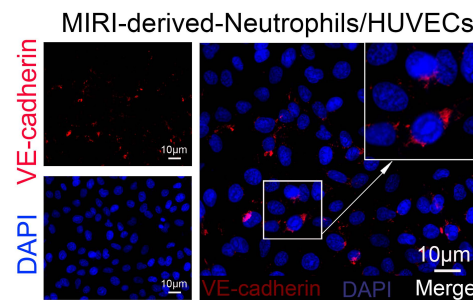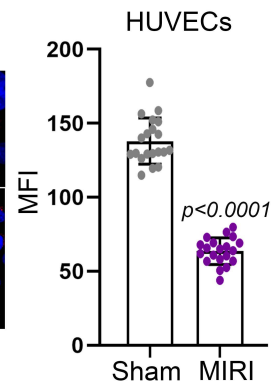

B

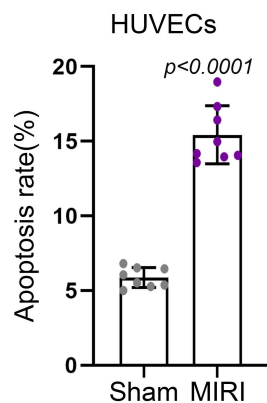

D

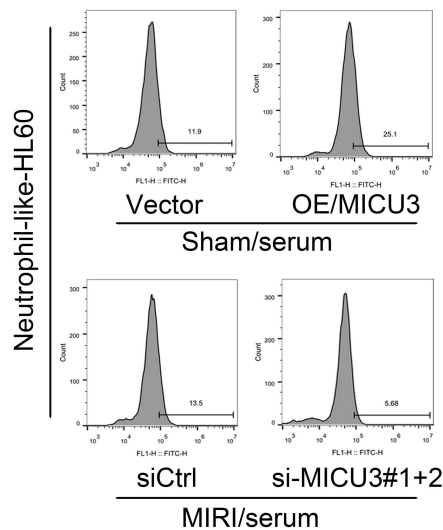

The proportion of FITC(+) Cells (%)

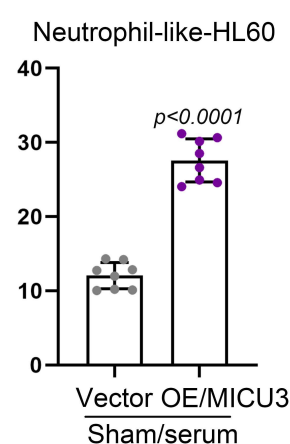

The proportion of FITC(+) Cells (%)

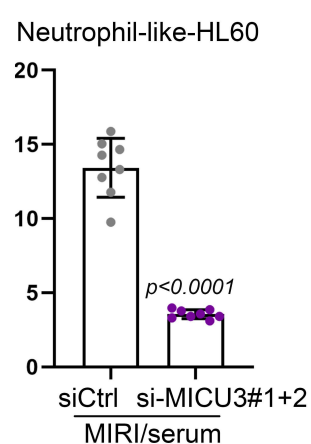

E

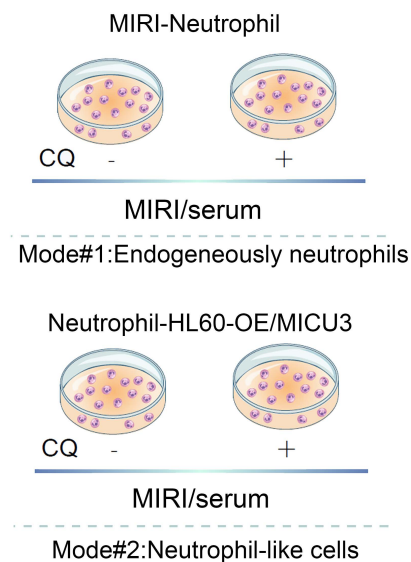

F

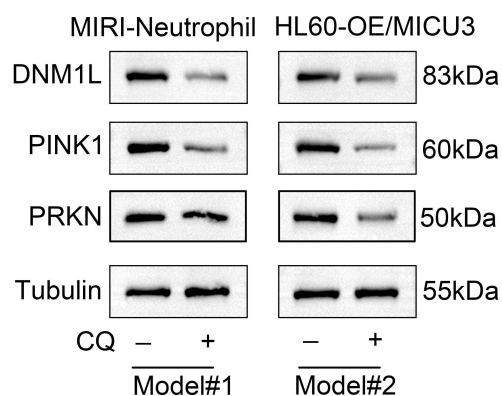

G

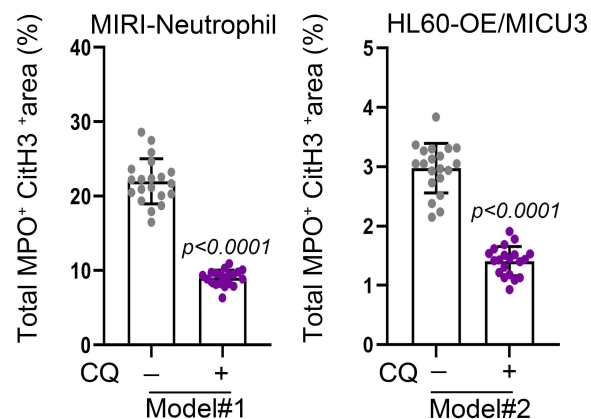

Supplement: Supplementary 1 — Figs. S1 to S3 [file research.0705.f1.zip › Figure S1.pdf]

# A

## Peripheral blood-Neutrophil

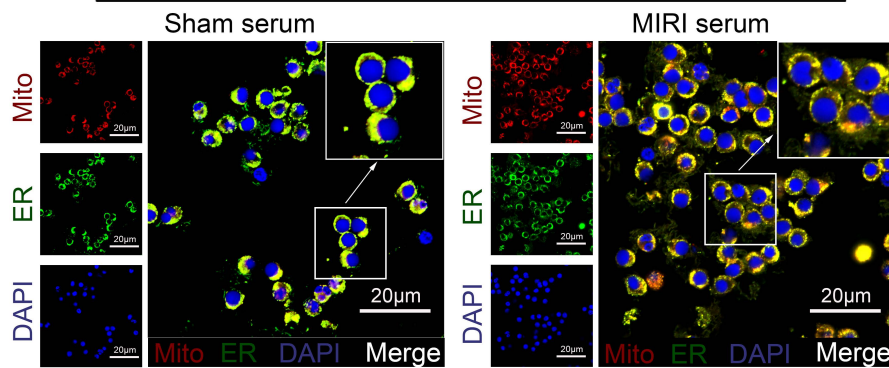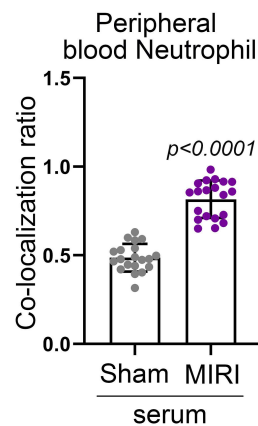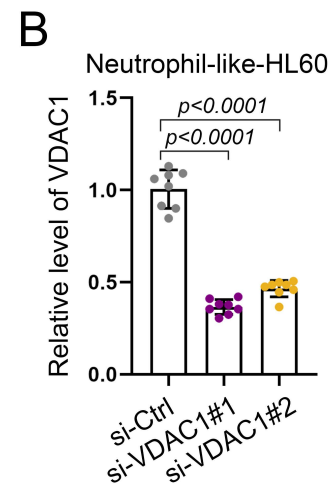

# C

## Neutrophil-like-HL60+MIRI/serum

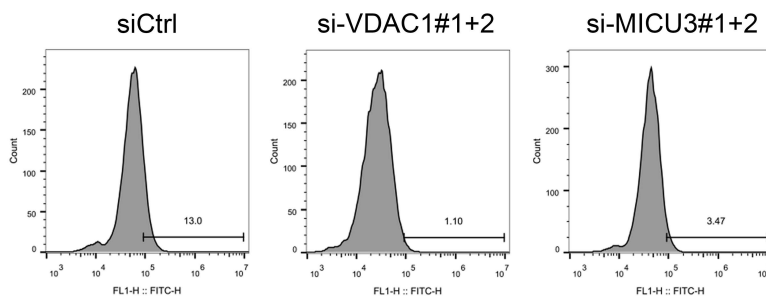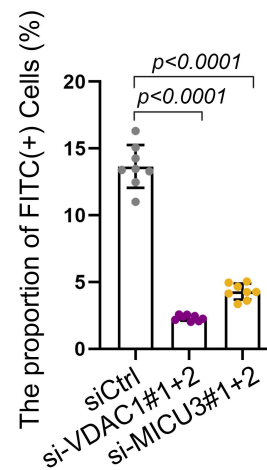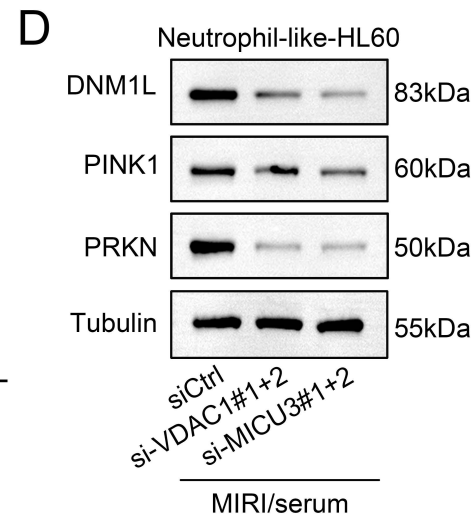

# E

## Neutrophil-like-HL60

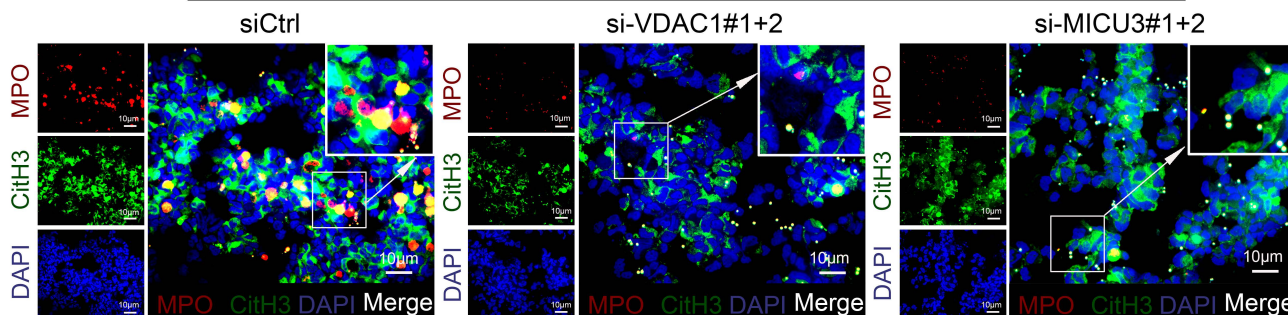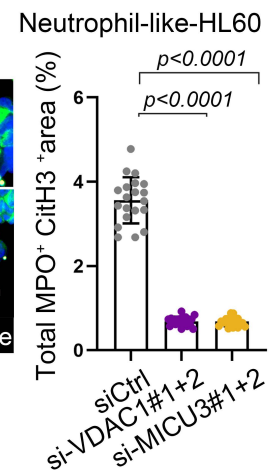

Supplement: Supplementary 1 — Figs. S1 to S3 [file research.0705.f1.zip › Figure S2.pdf]

A

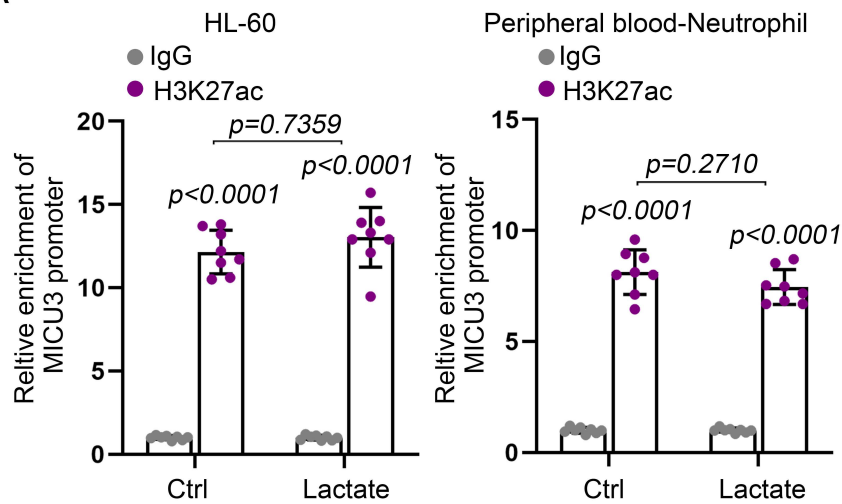

B

| ID          | position | Residue | PTMscores |
|-------------|----------|---------|-----------|
| NP_859074.1 | 480      | K       | 93.51%    |
|             | 486      | K       | 55.45%    |
|             | 500      | K       | 36.08%    |
|             | 493      | K       | 2.49%     |
|             | 267      | K       | 1.56%     |
|             | 469      | K       | 1.47%     |
|             | 274      | K       | 1.18%     |

C

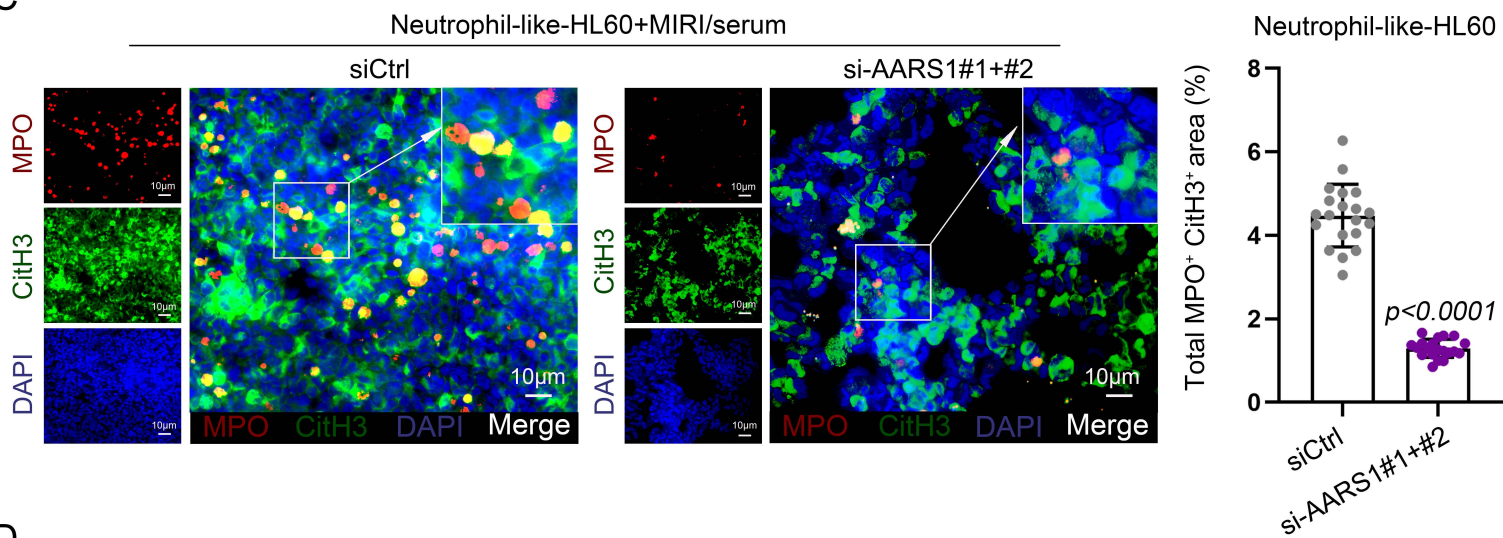

D

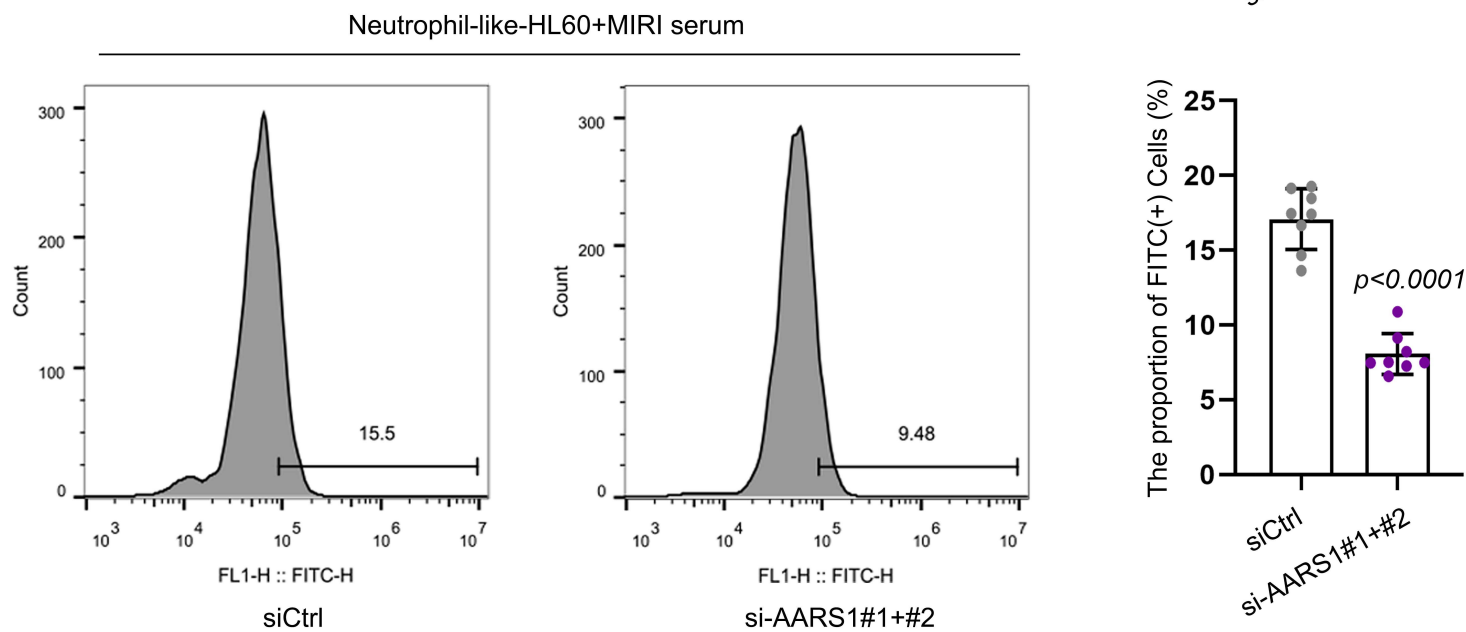

E

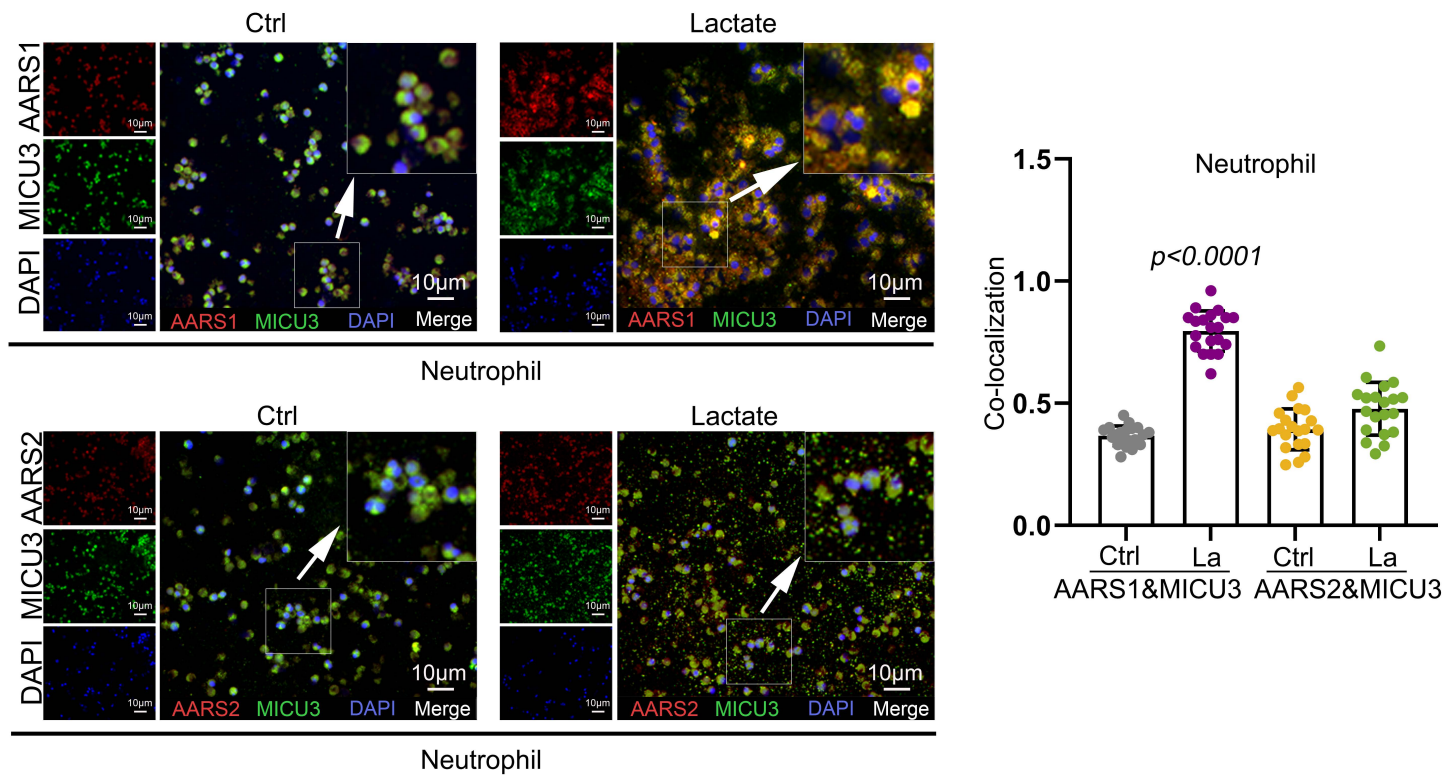

Supplement: Supplementary 1 — Figs. S1 to S3 [file research.0705.f1.zip › Figure S3.pdf]
